# A bile acid–GPBAR1 network supports anti-inflammatory and anti-fibrotic benefits of probiotics in colitis

**DOI:** 10.1080/19490976.2026.2645125

**Published:** 2026-03-18

**Authors:** Michele Biagioli, Cristina Di Giorgio, Silvia Marchianò, Benedetta Sensini, Ginevra Urbani, Eleonora Giannelli, Ginevra Lachi, Carmen Massa, Maria Rosaria Sette, Francesca Paniconi, Elva Morretta, Maria Chiara Monti, Angela Zampella, Eleonora Distrutti, Stefano Fiorucci

**Affiliations:** a University of Perugia, Medicine and Surgery, Department of Medicine and Surgery, Perugia, Italy; b University of Naples, Federico II, Pharmacy, Department of Pharmacy, Naples, Italy; c Azienda Ospedaliera di Perugia, SC di Gastroenterologia, Perugia, Italy

**Keywords:** Secondary bile acids, GPBAR1, intestinal fibrosis, IBD, probiotic, 3-oxo-DCA

## Abstract

Intestinal fibrosis is a severe complication of Crohn's disease for which therapy remains suboptimal. Probiotics are widely used in the treatment of intestinal inflammation, but all major guidelines do not recommend in favor of their use, with the exception of an 8-strains bacterial formula, which is recommended for the treatment of pouch inflammation in ulcerative colitis. Using this 8-strains formulation as a comparator, we characterized a 9-strains probiotic formulation enriched with *Lactobacillus rhamnosus* and *paracasei* in a mouse model of intestinal inflammation and fibrosis. Our findings demonstrated that while both formulations exerted similar protective effects in acute colitis, only the 9-strains probiotic attenuates inflammation and fibrosis in chronic colitis. Mechanistically, we found that the 9-strains formulation remodeled the microbiota composition and the structure of microbiota-derived secondary bile acids, leading to the selective enrichment of those bile acids that act as GPBAR1 agonists, including 3-oxo-DCA, which *in vitro* directly attenuates activation of intestinal fibroblasts. Confirming the role of this pathway, feeding Gpbar1^⁻/⁻^ mice with 9-strains probiotic formulation abrogates its beneficial effects on inflammation and fibrosis. These findings highlight the importance of microbial metabolites in shaping probiotic efficacy and support the development of probiotic formulations that target host–microbiota interactions through bile acid signaling.

## Graphical abstract

**Figure:**
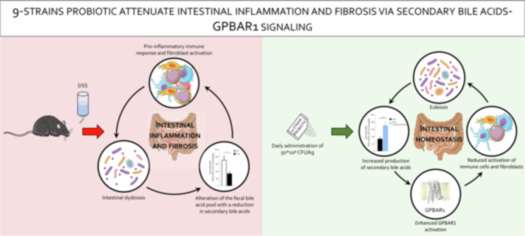

## Introduction

Inflammatory bowel diseases (IBDs), encompassing Crohn's disease (CD) and ulcerative colitis (UC), are chronic relapsing disorders characterized by dysregulated immune responses and persistent intestinal inflammation promoted by dysbiosis of the intestinal microbiota.^1^^,^^2^ Clinically, while UC is characterized by continuous mucosal inflammation confined to the colon, CD is a noncontinuous inflammation usually localized in the terminal ileum, although any region of the gastrointestinal tract can be involved. Phenotypically, CD is a fibrostenotic disorder characterized by transmural inflammation driven by dysfunctional extracellular matrix deposition and thickening of the bowel wall, leading to intestinal stenosis and fistulas in involved organs.^3‐5^ Mechanisms involved in fibrosis development and maintenance in CD patients are only partially identified, while treatment remains largely suboptimal, underscoring the need for novel therapeutic approaches.^6^

One functional consequence of intestinal dysbiosis is the perturbation of the structure of microbiota-derived metabolites.^7^ Secondary bile acids are a family of atypical steroids generated by microbial enzymes contributing to microbiota/host immune homeostasis. A reduction of secondary bile acids occurs in the majority of IBD patients and is a metabolomic signature of intestinal dysbiosis, contributes to immune dysfunction in both CD and UC and is a potential area of therapeutic intervention.^8‐10^

In contrast to primary bile acids, cholic acid (CA) and chenodeoxycholic acid (CDCA) generated in the liver from cholesterol metabolism, secondary bile acids are a large class of chemically different steroids,^11^ whose individual functions remain poorly defined.^12^^,^^13^ Various microbially depend modification of primary bile acids occur in the intestine, i.e. deconjugation (loss of the glycine and taurine groups), carried out by microbial bile salt hydrolases (BSH), oxidation of one of the hydroxyl groups, and 7α-dehydroxylation, that lead to the formation of secondary bile acids i.e. lithocholic and deoxycholic acid (LCA and DCA), and 7α/β epimerization with conversion of CDCA in ursodeoxycholic acid (UDCA).^13^^,^^14^ However, due to the bidirectional functionality of BSHs,^15^ the intestinal microbiota might operate the formation of a large number of amino-acid conjugates of primary and secondary bile acids, giving rise to the so-called microbial derived bile acids (MDBA).^15^ While over 200 amino acid-conjugated bile acids have been identified,^13^^,^^16^ most of these MDBA are found at very low quantities in the feces and body compartments, raising the questions over their real role in regulating microbial–host interactions. In addition to BSH-derived bile acids, bacterial oxidases also generate oxo-bile acids, including 3-, 7-, and 12-oxo derivatives of DCA and LCA, that regulate intestinal ecology and immunity.^17^ Excretion of some of these oxo-derivatives is reduced in IBD while their administration reverses intestinal inflammation in models of IBD.^17‐19^

Bile acids are endocrine mediators with high structure-related selectivity toward target receptors. Thus while all bile acids, because of their nonplanar conformation, lack activity toward classical hormone receptors, primary bile acids, CA and CDCA, activate the farnesoid-x-receptor (FXR) to regulate bile acid synthesis, while the secondary bile acids function as ligands for both nuclear receptors, vitamin D receptor (VDR) and RAR-related orphan receptor (ROR), and membrane receptors, such as the G protein-coupled bile acid receptor 1 (GPBAR1, also known as TGR5).^11^^,^^20^ Gene deletion and pharmacological studies have shown that GPBAR1 regulates the structure of the intestinal barrier and provides counterregulatory signals that attenuate inflammation, immune dysregulation and fibrosis in the intestine, and loss of function mutations of GPBAR1 are detected in UC and primary sclerosing cholangitis (a UC-associated disorder).^21‐24^

Probiotics are widely used as modulators of the structure and function of the gut microbiota. However, their clinical efficacy in IBD is limited, and their use is not generally recommended by current guidelines.^25‐27^ Probiotic formulations trialed in IBD patients vary substantially in terms of strains composition and amount of living cells^28^ administered, making it difficult to reach an effective comparison between currently available formulations. In general, however, while monostrain probiotics have shown some efficacy in improving patient's symptoms, they are only marginally effective in reducing intestinal inflammation and have no effect on clinically accepted end points, such as mucosal healing at endoscopy or nuclear magnetic resonance for CD.^29^ In contrast, a commercially available 8-strains combination of *Lactobacillus paracasei* subsp. *paracasei* (*L. paracasei* subsp. *paracasei*), *Lactobacillus plantarum* (*L. plantarum*), *Lactobacillus acidophilus* (*L. acidophilus*), *Lactobacillus delbrueckii* subsp. *bulgaricus* (*L. delbrueckii* subsp. *bulgaricus*), *Bifidobacterium longum* subsp. *longum* (*B. longum* subsp. *longum*), *Bifidobacterium breve* (*B. breve*), *Bifidobacterium longum* subsp. *infantis* (*B. longum* subsp. *infantis*), *and Streptococcus salivarius* subsp. *thermophilus* (*S. salivarius* subsp. *thermophilus*) is clinically approved for the treatment of UC-associated pouchitis,^30^ although it has no efficacy in treating CD.^26^

*Lactobacillus rhamnosus* (*L. rhamnosus*) and *Lactobacillus paracasei* (*L. paracasei*) are two well-characterized probiotics endowed with some health-promoting beneficial effects.^31‐35^ *Lactobacillus rhamnosus* (particularly the GG strain) is widely used in pediatric care for the prevention of intestinal enterocolitis in immature infants^36^ and is similar to *L. paracasei* has been trialed in IBD patients,^37‐39^ with some studies suggesting potential benefits in inducing and maintaining remission in UC patients.^40^

In the present study, we report the generation of a 9-strains probiotic formulation containing a high amount of *L. rhamnosus* and *L. paracasei*. We characterized its function in acute and chronic models of intestinal inflammation, fibrosis, microbiota composition, and detailed bile acid metabolic profiles. Furthermore, we explored the functional relevance of microbiota-derived secondary bile acids in modulating intestinal inflammation and fibrosis via GPBAR1 signaling, providing critical insights into the probiotic-bile acid-GPBAR1 axis. Our findings advance the understanding of probiotic-mediated modulation of the microbiota via the generation of secondary bile acid as a novel therapeutic strategy to manage inflammation and fibrosis in IBD.

## Materials and methods

### Acute and chronic mouse model of IBD

Wild-type C57BL/6J mice were obtained from Envigo (Italy). GPBAR1-deficient mice (Gpbar1^−/−^), which were originally developed on a C57BL/6 genetic background and kindly provided by Dr. Galya Vassileva (formerly at Schering-Plough Research Institute, Kenilworth, NJ, USA), were bred and maintained alongside their wild-type littermates in the certified animal facility of the University of Perugia. All animals were housed under standardized environmental conditions, including constant temperature (22 ± 1 °C) and a 12-h light/dark cycle, with *ad libitum* access to standard rodent chow and filtered tap water. Adult male mice (12–14 weeks old at the beginning of the experiment) were used for all experimental procedures.

We employed two experimental models of chemically induced colitis:

- *Acute colitis model.* Acute colitis was induced in C57BL/6 wild-type mice by administering 2% dextran sulfate sodium (DSS; molecular weight 40–50 kDa, Affymetrix, USA) in the drinking water for nine consecutive days.^41^ In the experimental groups requiring probiotic treatment, the formulations were administered daily from days 1 to 9 by oral gavage at a dose of 5 × 10¹⁰ bacteria/kg body weight/day.
- *Chronic colitis model.* To better investigate the fibrotic processes secondary to chronic intestinal inflammation, we induced chronic colitis in C57BL/6 wild-type mice as well as in Gpbar1^⁺/⁺^ and Gpbar1^⁻/⁻^ mice by administering 1.5% DSS in the drinking water for three cycles of 1 week each, interspersed with 15-d periods of regular water administration. The total duration of the experimental protocol was 57 d.^41^ This model, alternating DSS exposure and water, mimics the phases of relapse and remission that characterize the clinical course of human IBD. In the experimental groups requiring probiotic treatment, the formulations were administered daily from days 1 to 57 by oral gavage at a dose of 5 × 10¹⁰ bacteria/kg body weight/day.

The severity of colitis was measured each day for each mouse by analyzing body weight loss and occult blood and stool consistency. Each parameter was scored from 0 to 4, and the sum represents the Colitis disease activity index (CDAI).

Colitis disease activity index (CDAI) scoring criteria:

**Table:** 

Score	Weight loss (%)	Stool consistency	Blood
0	None	Normal	None
1	1–5	Soft but still formed	Occult blood
2	5–10	Very soft	Visible in stool (pink coloration)
3	10–20	Unformed stool, diarrhea	Traces of blood (bright red)
4	>20	Liquid stools adherent to the anus or anal blockage	Severe bleeding with fresh blood around the anus and abundant blood in feces

All experimental procedures were conducted in accordance with the European Directive 2010/63/EU for the protection of animals used for scientific purposes and were approved by the Animal Welfare Body of the University of Perugia, the Italian Ministry of Health, and the Istituto Superiore di Sanità (authorization no. 309–2022-PR). Mice were housed in specific pathogen-free conditions, and their overall health status was monitored daily by a facility veterinarian. At the experimental endpoint, the animals were euthanized before noon via deep anesthesia with sodium thiopental (200 mg/kg body weight, intraperitoneally).

Subsequently, blood was collected for complete blood count analysis, and the colon, small intestine, spleen, and mesenteric lymph nodes (mLNs) were harvested.

### Probiotics formulations

Two different formulations of probiotics were used in this study: a commercially available 8-strains probiotic and 9-strains probiotic. The 8-strains probiotic consists of a combination of *L. paracasei* subsp. *paracasei*, *L. plantarum*, *L. acidophilus*, *L. delbrueckii* subsp. *bulgaricus*, *B. longum* subsp. *longum*, *B. breve*, *B. longum* subsp. *infantis*, and *S. salivarius* subsp. *thermophilus* (brand name Vivomixx®, lot number 2331013, expiration date 11/2025; Mendes S.A., Lugano, Switzerland). The 9-strains probiotic consists of a combination of *L. paracasei*, Lactobacillus *rhamnosus* IMC 501 (*L. rhamnosus* IMC 501), *L. rhamnosus MC502*, *B. breve*, *Bifidobacterium lactis* (*B. lactis*), *L. acidophilus*, *L. plantarum*, *Lactococcus lactis* (*L. lactis*), and *Streptococcus thermophilus* (*S. thermophilus*). Individual bacterial strains used *in vitro* were provided by Mendes S.A. (Lugano, Switzerland), while the commercial formulation (Vivomixx neo9®, lot number 2433401, expiration date 05/2026, Mendes S.A., Lugano, Switzerland) was obtained from a pharmacy store. The two batches were maintained according to the manufacturer instructions until used.

### Histopathology

Colon samples (2–3 cm from the anus) were initially fixed in 10% formalin, embedded in paraffin, sectioned into 5 μm-thick slices, and stained with hematoxylin and eosin (H&E), Sirius red and Masson's trichrome for histopathological examination. All histological analyses were performed using an Olympus BX60 (Nikon DS-Ri2 camera) brightfield microscope.

Total histological score was calculated as the sum of the three individual parameter scores, yielding a final score ranging from 0 (no histological damage) to 12 (maximal injury). To ensure scoring accuracy, three representative images per mouse were evaluated, including one at 10× magnification and two at 20× magnification.

**Table:** 

Score	Inflammatory infiltration	Epithelial damage	Crypt architecture loss
0	No inflammatory cells	Intact epithelium	Normal crypt architecture
1	Mild infiltration restricted to the mucosa	Focal areas of epithelial cell loss	Mild crypt distortion or rare crypt loss
2	Moderate infiltration extending to the submucosa	Multiple regions of erosion or denudation	Focal crypt loss with moderate disorganization
3	Severe infiltration with distortion of tissue architecture	Extensive ulceration	Diffuse loss of crypts and severe architectural disruption
4	Extensive transmural infiltration with mucosal destruction	Complete loss of epithelial layer and mucosal integrity	Complete crypt loss and mucosal collapse

Wall thickness was assessed to evaluate colon fibrosis and structural changes in acute and chronic mouse models of colitis. Wall thickness was evaluated in H&E-stained sections imaged at 20× magnification. Muscularis propria thickness was measured seven to eight times throughout the entire colon circumference. The final value was obtained as the average of the measurements taken.

Colon fibrosis score was evaluated in Sirius red-stained sections imaged at 20× magnification. For quantification, the images were analyzed using ImageJ software. The stained area was quantified as the percentage of red-stained (positive for collagen) area relative to the total area of each section. At least four to five random fields per section were quantified. The final value was obtained as the average of the measurements taken.

For the assessment of fibrosis, the tissue sections were additionally evaluated using Masson's trichrome staining. Colon fibrosis score was evaluated in Masson's trichrome-stained sections imaged at a 20× magnification. The stained area was quantified as the percentage of blue-stained (positive for collagen) area relative to the total area of each section. At least four to five random fields per section were quantified. The final value was obtained as the average of the measurements taken.

Immunofluorescence analysis was performed on paraffin-embedded mouse colon sections. Antigen retrieval was conducted by heating the tissue sections in sodium citrate buffer (pH 6.0) at 95 °C for 90 min. After retrieval, the slides were permeabilized with PBS containing 0.1% Triton, followed by incubation in blocking buffer (PBS 1× with 10% horse serum and 1% BSA) for 1 h at room temperature. The sections were then incubated overnight at 4 °C with primary antibodies against E-CADHERIN (1:100) (GTX100443, Genetex, Irvine, CA, USA). The next day, after three washes with PBS 1× containing 0.1% Tween 20 (PBST), the sections were incubated with the secondary antibody Alexa Fluor™ 568 A-11011 (1:1,000) (Invitrogen, Thermo Fisher Scientific, Waltham, MA, USA) for 1 h at room temperature in the dark. Following three additional washes with PBST, the nuclei were counterstained with DAPI 1× for 1 min in the dark, and the reaction was terminated with a final PBS 1× wash for 5 min. The slides were then mounted with ProLong Glass Antifade Mountant (P36980) (Invitrogen, Carlsbad, CA), sealed with nail polish, and observed under an Olympus BX60 fluorescence microscope. The quantification of immunofluorescence was performed using the Fiji software (Fiji Is Just ImageJ) as described in a previous work.^17^

### AmpliSeq transcriptome

Total RNA was extracted from the mouse colon sample using the MagMAX™ mirVana™ Total RNA Isolation Kit (Thermo Fisher Scientific, Waltham, MA) according to the manufacturer's instructions and processed via the KingFisher™ Flex automated purification system (Thermo Fisher Scientific, Waltham, MA). The quality and concentration of the extracted RNA were evaluated using the Qubit® RNA HS Assay Kit on a Qubit 3.0 fluorometer, followed by confirmation through agarose gel electrophoresis. Library preparation was performed using the Ion AmpliSeq™ Transcriptome Mouse Gene Expression Core Panel and the Chef-Ready Kit (Thermo Fisher Scientific), following the manufacturer's instructions. Barcoded libraries were employed for the generation of Template-Positive Ion Sphere™ Particles (Thermo Fisher Scientific, Waltham, MA), which were loaded onto Ion 540™ Chips (Thermo Fisher Scientific, Waltham, MA). Sequencing was conducted on an Ion S5™ System, and data acquisition was managed through Torrent Suite™ Software v6 (Thermo Fisher Scientific). Differential gene expression analysis was performed using the TAC software (version 4.0.2), applying a fold-change cutoff of <–2 or >+2 and a *p* value < 0.05.

### Isolation of intestinal *lamina propria* cells

At the conclusion of the experiments, the colons were excised and carefully rinsed to remove residual fecal material. *Lamina propria* cells were then isolated using the Lamina Propria Dissociation Kit (Miltenyi following the manufacturer's recommended protocol.

When required by the experimental design, colonic *lamina propria* cells were further separated by magnetic cell sorting with CD45 MicroBeads (Miltenyi Biotec) in accordance with the manufacturer's protocol, yielding both the positively selected CD45⁺ leukocyte fraction and the negatively selected CD45^−^ nonimmune fraction.

### Flow-cytometry

Cells isolated from the colonic *lamina propria* were examined via spectral flow cytometry using a Cytek® Northern Lights™ cytometer equipped with violet (405 nm), blue (488 nm), and red (640 nm) excitation lasers, facilitating high-dimensional fluorescence analysis via complete spectral acquisition. Instrument configuration and spectral unmixing protocols were optimized with reference controls supplied by the manufacturer. Data obtained from flow cytometry experiments were processed and analyzed utilizing FlowJo software (Tree Star, Ashland, OR, USA).

Two distinct staining protocols, employing different antibody panels, were performed on cells isolated from the colonic *lamina propria*.

**Table:** 

STAINING1				
Antigen	Clone	Fluorocrome	Manufacturing company	Catalog number
CD45	30-F11	APC	Invitrogen	MCD4505
CD3	145-2C11	PerCP-Cy5.5	Invitrogen	45-0031-82
CD49b	DX5	APC-eFLuor780	Invitrogen	47-5971-82
CD4	GK1.5	NovaFluor Red 700	eBioscience	M001T02R03-A
CD8a	53-6.7	SB702	Invitrogen	67-0081-82
B220	RA3-6B2	SB600	Invitrogen	63-0452-82
CD11b	M1/70	FITC	BioLegend	101206
GR1 (Ly-6G/Ly-6C)	RB6-8C5	BV510	BioLegend	108438
CD38	90	PE-Cy7	Invitrogen	25-0381-82
CD206 (MMR)	MR6F3	PE	Invitrogen	12-2061-82

CD45^+^ cells labeled with this staining panel were subsequently analyzed using FlowJo software, initially employing t-distributed stochastic neighbor embedding (tSNE) for dimensionality reduction, followed by unsupervised clustering through the FlowSOM plugin, resulting in the identification of eight distinct cell populations.

**Table:** 

STAINING_2_				
Antigen	Clone	Fluorocrome	Manufacturing company	Catalog number
CD11b	M1/70	APC-eFluor780	Invitrogen	47-0112-82
GR1 (Ly-6G/Ly-6C)	RB6-8C5	SB600	Invitrogen	63-5931-82
IL-6	MP5-20F3	PerCP-eFLuor 710	Invitrogen	46-7061-82

Cells isolated from the colonic *lamina propria* and labeled with this staining panel were analyzed with FlowJo software using conventional flow cytometry, and the gating strategy was established based on the fluorescence minus-one (FMO) control methodology.

### Gut microbiota analysis

Microbial DNA was isolated from fecal material collected from the colonic contents of mice using the PureLink™ Microbiome DNA Purification Kit (Thermo Fisher Scientific, Waltham, MA) in accordance with the manufacturer's instructions. The DNA concentration was assessed using the Qubit™ 3.0 fluorometer following the recommended protocol.

Barcoded library preparation and template generation were performed using the Ion 16S Metagenomics Kit in combination with the Ion 510™, 520™, and 530™ Kit - Chef following the manufacturer's guidelines. Sequencing was conducted on the Ion S5™ platform. Taxonomic profiling and annotation were carried out using the Ion Reporter™ software (version 5.18.4.0). Microbial identification at the family, genus, and species levels was achieved through alignment against the curated Greengenes v13.5 reference database.

### Bile acid determinations in murine fecal samples

Conjugated and unconjugated bile acids were measured in plasma and colon feces. Plasma samples were treated as follows: 50 µL of each sample were extracted with 200 µL of MeOH through shaking and sonication. Following centrifugation (10,000 × *g* for 10 min), equal volumes of each extract were dried under vacuum and resuspended in a mixture comprising H_2_O and MeOH (50:50 vol/vol). Colon feces were first lyophilized, then weighed and extracted at a concentration of 50 mg of feces per mL of MeOH through shaking and sonication. Following centrifugation (10,000 × *g* for 10 min), equal volumes of each extract were dried under vacuum and resuspended in a mixture of H_2_O and MeOH (50:50 vol/vol) at a final concentration of 25 mg of feces per mL of buffer.

Conjugated and unconjugated BA standards were prepared in MeOH and they were mixed in H_2_O/MeOH (50:50 vol/vol) to obtain calibration curves spanning from 1.5 to 50 nM. Calibration curves and plasma and feces extracts were then analyzed through liquid chromatography‒mass spectrometry (UHPLC‒MS) on a Tribrid Orbitrap mass spectrometer coupled to a Vanquish Flex ultra-high pressure liquid chromatography (UHPLC) system (Thermo Fisher Scientific, Bremen). BA was separated on a Luna Omega Polar C18 column (1.6 μm, 100 Å, 100 × 2.1 mm, Phenomenex) at a flow rate of 400 µL/min (50 °C) with the following gradient: 2 min at 45% B, 2 min to 11 min to 60% B, then held at 60% B until 15 min, 15 min to 23 min from 60% B to 80% B, then 80% B to 95% B for 1 min (A: H_2_O, 5 mM ammonium acetate, 0.01% FA; B: 80% MeOH, 20% CH_3_CN, 0.01% FA).

The mass spectrometer was operated in MS1 profiling negative scan mode. Full-scan MS spectra were acquired in the Orbitrap with the following settings: scan range, 350–700 m/z; full-scan automatic gain control (AGC) target at 25% at 120,000 resolution; and maximum injection time mode, “Auto.” Source parameters were set as follows: spray voltage at 3,000 V; sheet gas at 50 (Arb); auxiliary gas at 10 (Arb); ion transfer tube temperature at 325 °C; and vaporizer temperature at 350 °C. For each BA, the extracted ion chromatogram was obtained from the raw files through SkyLine software, and each peak area was measured.

### Human data

The metabolomic data derived from samples of healthy donors (HS, *n *= 26), UC patients (UC, *n *= 30), and Crohn's disease patients (CD, *n *= 50) were retrieved from the Inflammatory Bowel Disease Multi'omics Database (IBDMDB).^8^

### Proteomic analysis

Proteomic analysis was carried out on 9-strains probiotic and on each of the following individual bacterial strains: *B. animalis* (subsp. *Lactis*), *B. breve*, *S. thermophilus*, *Lactococcus lactis*, *L. rhamnosus*, *L. rhamnosus* (*ICM501*), *L. paracasei* (*ICM502*), *L. plantarum*, and *L. acidophilus*. Proteins extraction from lyophilized bacteria was achieved through BugBuster Master Mix (Merck Millipore), as reported by the manufacturer. Briefly, 10 mg of lyophilized bacteria were treated with 100 µL of BugBuster Master Mix supplemented with a cocktail of endogenous proteases (final concentration 1×, GeneSpin). Lysis was achieved by shaking the samples for 30 min at 600 rpm and 25 °C and then centrifuging them for 30 min (14,800 rpm, 4 °C). Finally, the supernatant was collected, and the protein concentration was determined through Bradford assay. Subsequently, 5 µg of proteins were treated with Laemmli sample buffer (100 mM Tris–HCl pH 6.8, 4% p/V SDS, 0.2% p/V bromophenol blue, 20% vol/vol glycerol, 2% vol/vol β-mercaptoethanol), boiled at 95 °C for 5 min, and loaded on a 12% polyacrylamide SDS-PAGE gel. SDS‒PAGE was performed at 150 V in Tris–glycine–SDS buffer, then the gels were fixed and stained with Coomassie G-250 (Bio-Rad), and scan images were obtained (ChemiDoc^TM^ Imaging System, Bio-Rad). Subsequently, gel bands were excised from the gels and submitted to *in situ* tryptic digestion as previously reported.^42^

About 5 μL of each digest were analyzed on a Tribrid Orbitrap mass spectrometer coupled to a VanquishNeo ultra-high pressure liquid chromatography (UHPLC) system (ThermoFisher Scientific) equipped with an EASY-Spray PepMAP^TM^ Neo C18 column (1,500 bar, 2 μm, 100 Å, 75 μm × 500 mm). Peptide elution was performed at 270 nL/min with the following gradient: from 3% B to 45% B in 45 min (A: H_2_O, 0.1% FA; B: 80% CH_3_CN, 20% H_2_O, 0.1% FA). The mass spectrometer was operated in data-dependent acquisition mode. Full-scan MS spectra were acquired with the following settings: scan range 375–1,500 m/z; normalized full-scan automatic gain control (AGC) target set at 100% at 240,000 resolution; and maximum injection time of 50 ms. MS2 spectra were generated with HCD (normalized collision energy of 30%). Normalized AGC target was set as 300%, with a maximum injection time of 35 ms. The raw files obtained were then analyzed through Proteome Discoverer (version 3.1.0.638). Spectra were searched by Sequest against protein databases specific to each bacterial strain, using the following parameters: trypsin digestion; maximum of two missed cleavages; cysteine carboxyamidomethylation as a fixed modification; and methionine oxidization as a variable modification. Precursor mass and fragment mass tolerances were respectively set at 10 ppm and 0.6 Da, respectively.

### Probiotic culture for bile acid metabolism analysis

Probiotic mixture Vivomixx neo9® was cultured in liquid brain heart infusion (BHI) medium at 37 °C with agitation for 72 h under anaerobic conditions, following inoculation at an initial density of 10⁷ CFU/mL. Anaerobiosis was generated inside a culture jar using a GENbox anaer (ref. 96 124, bioMérieux). Bile acids were added to the culture medium at a final concentration of 100 µM. We cultured the bacteria in the presence of the following bile acid concentrations: CA (50 µM) + CDCA (50 µM); tCA (50 µM) + tCDCA (50 µM); LCA (50 µM) + DCA (50 µM); tLCA (50 µM) + tDCA (50 µM); and a mixed condition containing CA (12.5 µM), tCA (12.5 µM), CDCA (12.5 µM), tCDCA (12.5 µM), LCA (12.5 µM), tLCA (12.5 µM), DCA (12.5 µM), and tDCA (12.5 µM). After the incubation period, the bacterial cells were pelleted via centrifugation, and the resulting supernatant and bacterial cells were collected for bile acid analysis as described in the corresponding Materials and Methods section.

### Bile acid determinations in bacteria culture medium

Conjugated and unconjugated bile acids were measured in the 9-strains probiotic culture medium 72 h after BAs supplementation. The samples were treated as follows: 200 μL of each sample were extracted with 800  μL of MeOH through shaking and sonication. Following centrifugation (10,000 × *g* for 10 min), 5 µL was run on the LC‒MS system. Chromatographic separation was carried out on the UHPLC‒MS system Xevo TQ absolute mass spectrometer from Waters equipped with Acquity I class Plus pumps and an autosampler. The mixture was separated on a Luna Omega Polar C18 1.6 µm, as reported above. The source parameters for BAs were as follows: soft transmission mode disabled, capillary (kV) 2.50; cone (V) 88.00; source offset (V) 30.0; source temperature (°C) 150; desolvation temperature (°C) 600; cone gas flow (L/h) 150; desolvation gas flow (L/h) 500; collision gas flow (mL/min) 0.15; nebulizer gas flow (L/h) 300; LM 1 resolution 2.8 and HM 1 resolution 14.2. Each BA has been identified by comparison with its pure standard retention time in MRM mode using the Target Link by Waters.

### Luciferase reporter assay

The luciferase reporter assay is performed over a 4-d period. On day 1, HEPG2 or HEK293T cells were seeded in 24-well plates at a density of 7.5 × 10⁴ cells/well and 2.5 × 10⁴ cells/well, respectively. On the second day, the cells were transiently transfected, with separate transfection mixtures prepared for each receptor. HEPG2 cells were transfected with 200 ng of the reporter vector containing the response element p(hsp27)-TK-LUC, 100 ng of pSG5-FXR, 100 ng of pSG5-RXR, and 100 ng of pGL4.70, which encodes the Renilla luciferase gene. For GPBAR1 activation, HEK293T cells were transfected with 200 ng of the reporter vector pG29-CRE-LUC, 150 ng of pcmvsport-hTGR5, or 150 ng of pGL4.70. Transfections were carried out using FuGENE® HD (Promega) according to the manufacturer's protocol to facilitate the delivery of plasmid DNA into the cells. Twenty-four hours post-transfection, HEPG2 cells were stimulated with 10 μM CDCA as a positive control and with 10 μM different BAs compounds to evaluate their agonistic effects on the FXR/RXR receptor. Similarly, HEK293T cells were treated with 10 μM of TLCA, and with 10 μM Bas to assess GPBAR1 receptor activation. After 24 h of stimulation, the cells were lysed in 100 μL of lysis buffer to prepare the samples for the Dual-Luciferase® Reporter Assay System (Promega), which allows for the measurement of both firefly and Renilla luciferase activities via luminescence.

### Coculture

The coculture setup involved the use of selected cell lines: human small intestinal fibroblasts (IM-HSIF), colorectal adenocarcinoma epithelial cells (HT29), and human monocytes (U937). They were maintained in culture under distinct conditions: IM-HSIF (Innoprot) and U937 (ATTC) cells were cultured in RPMI (Euroclone) enriched with 10% fetal bovine serum, 1% L-glutamine, and 1% penicillin/streptomycin, while HT29 cells were cultured in D-MEM (Euroclone) supplemented with the same additives. All the cultures were maintained in a humidified environment with 5% CO_2_ at 37 °C.

For the experimental protocol, Nunc™ polycarbonate cell culture inserts in multiwell plates (Thermo Fisher) were used to mimic an inflammatory niche resembling the IBD condition. The experimental timeline was set to 1 week.

On the first day, only HT29 cells (4 × 10⁴ cells in 600 µL per insert) were seeded into the inserts of each well to allow the formation of a confluent epithelial monolayer. To prevent the insert membrane from drying out, additional medium was added to the lower part of the chamber (700 µL per well). The medium was refreshed every other day until the stimulation phase.

On the second day, IM-HSIF (1 × 10⁵) and U937 (2 × 10⁵) cells were seeded simultaneously (700 µL per well), but in a separate plate from that containing the epithelial cells.

On the third day, the media of the HSIF and U937 cocultures were replaced and supplemented with phorbol 12-myristate 13-acetate (PMA, 100 nM per well). The cells were then incubated for 48 h to allow the differentiation of monocytes into activated macrophages. During this resting period, all the cultures were routinely monitored.

On the fourth day, the cells were subjected to serum starvation. After 24 h, they were stimulated with TNF-α (25 ng/mL) and LPS (100 ng/mL), either alone or in combination with 3-OXO-DCA (10 µM), for 3 d. At this stage of the protocol, the coculture was established by placing the HT29-containing inserts into the wells containing the HSIF and differentiated macrophages. Prior to assembling the co-culture, each cell type was prestimulated individually under the same conditions using its respective culture medium. The eventual mixing of media types was not problematic for the experimental setup.

After 72 h, U937 and IM-HSIF cells were collected and separated through magnetic cell sorting with CD45 MicroBeads (Miltenyi Biotec), in accordance with the manufacturer's protocol, to analyze inflammation (U937 cells) and fibrosis (IM-HSIF cells) and extracted for qPCR analysis using the Direct-zol™ RNA MiniPrep Kit with Zymo-Spin™ IIC Columns (Zymo Research, Irvine, CA) according to the manufacturer's instructions.

### RNA isolation and qRT-PCR

Total RNA was extracted from both the CD45⁺ and CD45⁻ cell fractions isolated from the colonic *lamina propria* and from the U937 and IM-HSIF human cell lines for the coculture experiment using the MagMAX™ mirVana™ Total RNA Isolation Kit (Thermo Fisher Scientific, Waltham, MA), according to the manufacturer's instructions, and processed with the KingFisher™ Flex automated purification system (Thermo Fisher Scientific, Waltham, MA). A total of 1 μg of purified RNA from each cell sample was then reverse transcribed into cDNA using the FastGene Scriptase Basic Kit (Nippon Genetics, Mariaweilerstrase, Düren, Germany) in a reaction volume of 20 μL. Subsequently, 50 ng of cDNA were subjected to amplification in a 20  μL reaction volume containing 200 nM of each primer and 10 μL of PowerUp™ SYBR™ Green Master Mix (Thermo Fisher Scientific, Waltham, MA). Reactions were performed in triplicate using a QuantStudio™ 7 Flex Real-Time PCR System (Applied Biosystems, Foster City, CA) under the following thermal profile: initial denaturation at 95 °C for 2 min, followed by 40 cycles of 95 °C for 3 s and 60 °C for 30 s. Relative mRNA expression levels were calculated using the 2^(−Δ*Ct*) method. The sequences of the primers (forward and reverse) were as follows:

**Table:** 

Mice genes		
Gene	Primer sequence	
	Forward	Reverse
*Tbp*	TCTACCGTGAATCTTGGCTGT	ATGATGACTGCAGCAAATCG
*Hprt*	CAGTCCCAGCGTCGTGATTA	TTTTCCAAATCCTCGGCATA
*Polr2a*	TTGTATCCGTACCCACAGCA	GCTCCCCATTCTCCACTACA
*Tnf-α*	GCCTCTTCTCATTCCTGCTT	GAGGCCATTTGGGAACTTCT
*Ccl2*	AAGAGGATCACCAGCAGCAG	TCTGGACCCATTCCTTCTTG
*iNos*	GGAGGTGACCATGGAGCATC	CACCCACCTCCAGTAGCATG
*αSma*	AGAGCTACGAACTGCCTGAC	TAGGTGGTTTCGTGGATGCC
*Col3a1*	GCACAGCAGTCCAACGTAGA	ATTTGACATGGTTCTGGCTTCC
*Col1a1*	TGACTGGAAGAGCGGAGAGT	AGACGGCTGAGTAGGGAACA

**Table:** 

Human genes		
Gene	Primer sequence	
	Forward	Reverse
*HPRT*	AAGGGTGTTTATTCCTCATGGA	TTGATGTAATCCAGCAGGTCAG
*POLR2A*	TTGGGGAGATTGAGTCCAAG	ATCTGGGTGGCAGGTTCTC
*IL-6*	AAAGAGGCACTGGCAGAAAA	TCAAACTCCAAAAGACCAGTGA
*TNFα*	AGCCCATGTTGTAGCAAACC	TGAGGTACAGGCCCTCTGAT
*IL-1β*	GTGGCAATGAGGATGACTTG	GGAGATTCGTAGCTGGATGC
*CCL2*	TAGCAGCCACCTTCATTCCC	CTGCACTGAGATCTTCCTATTGG
*αSMA*	GTGTTCCCGTCCATCGTG	CTCTTGCTCTGAGCCTCGTC
*TIMP1*	AGACGGCCTTCTGCAATTC	TCATAACGCTGGTATAAGGTGGT

### Statistical analysis

We first performed the Kolmogorov‒Smirnov test for normal distribution. One-way ANOVA or unpaired Student's *t* test was used for statistical comparisons (*p < 0.05). All analyses were performed using the Prism 8.0 software (GraphPad).

## Results

### Effects of the probiotics formulations in mouse model of acute colitis

**Figure 1.**
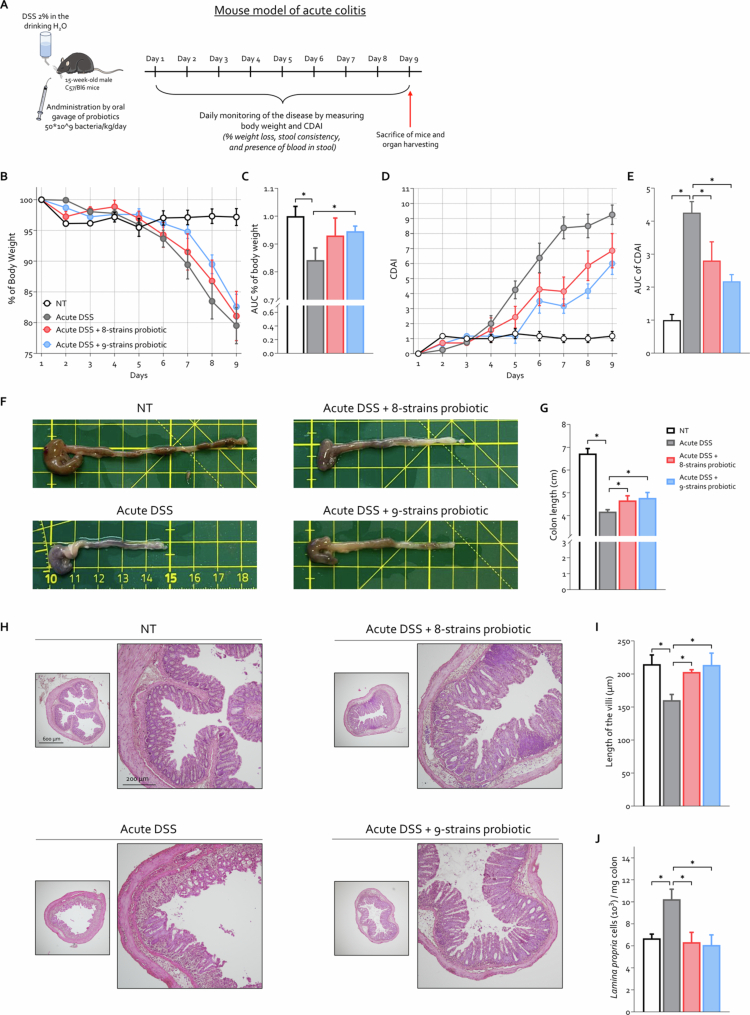
Comparison of probiotic efficacy in an acute DSS-induced colitis model. C57BL/6 male mice were treated with DSS alone or in combination with 8-strains probiotic or 9-strains probiotic (50 × 10^9^ bacteria/kg/day). (A) Schematic representation of the experimental protocol used to induce acute DSS-induced colitis. (B) Body weight changes and (C) corresponding areas under the curve (AUC) normalized to those of the NT experimental group. (D) Colitis Disease Activity Index (CDAI) and (E) AUC normalized to those of the NT experimental group. (F) Representative macroscopic images of the colons from each experimental group. (G) Colon length (cm). (H) Representative hematoxylin and eosin (H&E)-stained sections of the colon (original magnification 10× and 20×). (I) Quantification of colonic villus length. (J) Total number of leukocytes in the colonic *lamina propria* as a measure of inflammation severity. Graphs show mean ± SEM of 6 NT, 8 DSS, 7 DSS+ 8-strains probiotic, and 8 DSS+ 9-strains probiotic mice. Statistical analysis was performed using the Kolmogorov–Smirnov test for normality followed by one-way ANOVA (*p < 0.05).

We first investigated the effect of the novel 9-strains probiotic formulation in comparison with those of a commercially available 8-strains probiotic in a murine model of acute colitis. In this experimental set, colitis was induced by administering 2% DSS dissolved in the drinking water for nine consecutive days. The probiotic formulations were administered daily by oral gavage at a dose of 50 × 10^9^ bacteria/kg body weight from days 1 to 9 (Figure 1A). Disease progression was monitored daily through measurements of body weight and the Colitis Disease Activity Index (CDAI). The data obtained demonstrated that both probiotic formulations significantly alleviated the clinical signs and symptoms of colitis (Figure 1B–E) and also reduced both macroscopic and microscopic colon damage (Figure 1F–I). Furthermore, disease induction resulted in a notable increase in infiltrating cells in the colonic *lamina propria* (Figure 1J), an effect that was also reversed by the administration of both probiotic formulations. Collectively, these findings indicate that probiotic treatment effectively ameliorates the clinical manifestations and tissue damage associated with DSS-induced acute colitis.

### Probiotic intervention attenuates chronic colitis and fibrosis in a DSS-induced mouse model

**Figure 2.**
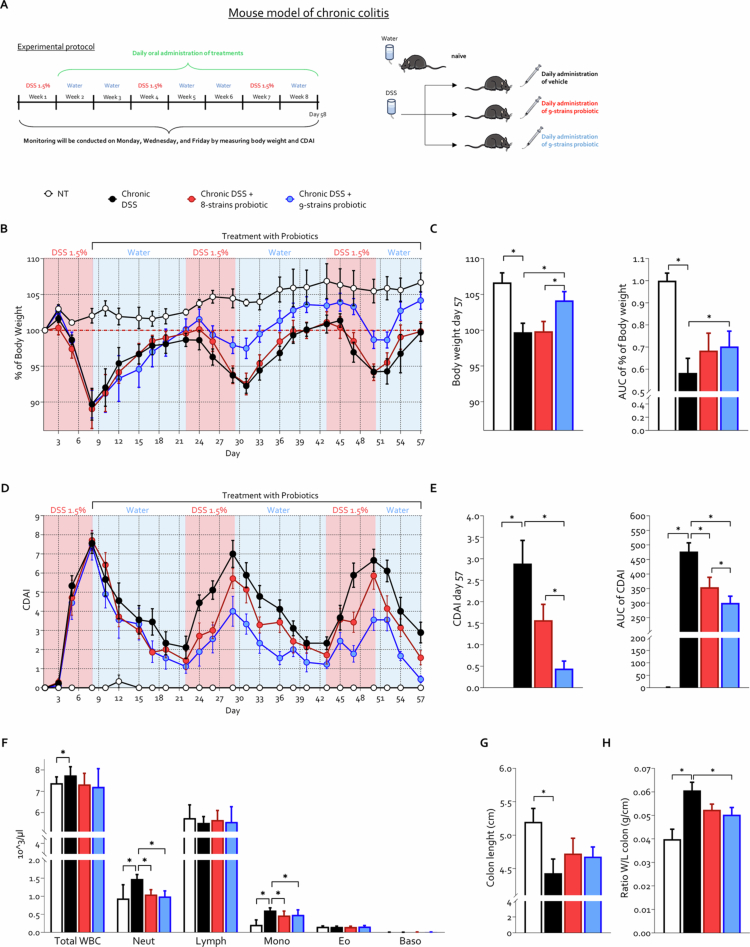
Differential effects of 8-strains and 9-strains probiotic formulations in chronic DSS-induced colitis. C57BL/6 male mice were treated with three cycles of DSS alone or in combination with 8-strains probiotic or 9-strains probiotic (50 × 10^9^ bacteria/kg/day). (A) Schematic representation of the experimental protocol used to induce chronic DSS-induced colitis. (B) Body weight changes and (C) corresponding AUC normalized to NT experimental group. (D) Colitis Disease Activity Index (CDAI) and (E) AUC normalized to NT experimental group. (F) Analysis of immune cell populations in peripheral blood (complete blood count) collected on the day of sacrifice. (G) Colon length (cm) and ratio of weight versus colon length (g/cm). Graphs show mean ± SEM of 5 NT, 10 DSS, 8 DSS+ 8-strains probiotic, and 10 DSS+ 9-strains probiotic mice. Statistical analysis was performed using the Kolmogorov–Smirnov test for normality followed by one-way ANOVA (*p < 0.05).

IBDs are chronic conditions characterized by alternating phases of remission and relapse. The persistent inflammation typical of these disorders frequently results in intestinal fibrosis, a severe complication for which effective treatments are currently lacking.^5^ Based on this unmet need, we assessed the efficacy of the two probiotic formulations in a chronic murine model of colitis, characterized by alternating DSS administration and water periods,^41^ thereby mimicking the typical cycles of relapse and remission observed in human disease and inducing the development of intestinal fibrosis (Figure 2A). Data on body weight variation and CDAI scores revealed differential efficacy between the two probiotic formulations in the chronic colitis model, in contrast to what was observed in the acute setting (Figure 1). Specifically, the 8-strains probiotic showed no effect on body weight modulation and exerted only a modest therapeutic benefit on the CDAI, as evidenced by a statistically significant reduction in the area under the curve (AUC) of the CDAI score compared to the mice treated with DSS alone (Figure 2B–E). In contrast, the 9-strains probiotic exerted a marked therapeutic effect, significantly attenuating both weight loss and CDAI scores relative to those of the DSS-treated controls (Figure 2B–E). Notably, a statistically significant difference was also observed between the two probiotic-treated groups with respect to both body weight changes and CDAI scores, further supporting the superior efficacy of the 9-strains formulation (Figure 2C and E). Induction of chronic colitis resulted in a significant increase in circulating total white blood cells (WBC),(WBCs), particularly neutrophils and monocytes, which is indicative of systemic inflammation (Figure 2F). Treatment with both probiotic formulations significantly reduced the circulating neutrophil and monocyte counts having a similar effect. At the colonic level, disease induction was associated with colon shortening and an increased colon weight-to-length ratio, both indicative of intestinal inflammation (Figure 2G, H). Notably, only treatment with the novel 9-strains probiotic formulation significantly reduced the colon weight-to-length ratio (Figure 2H).

**Figure 3.**
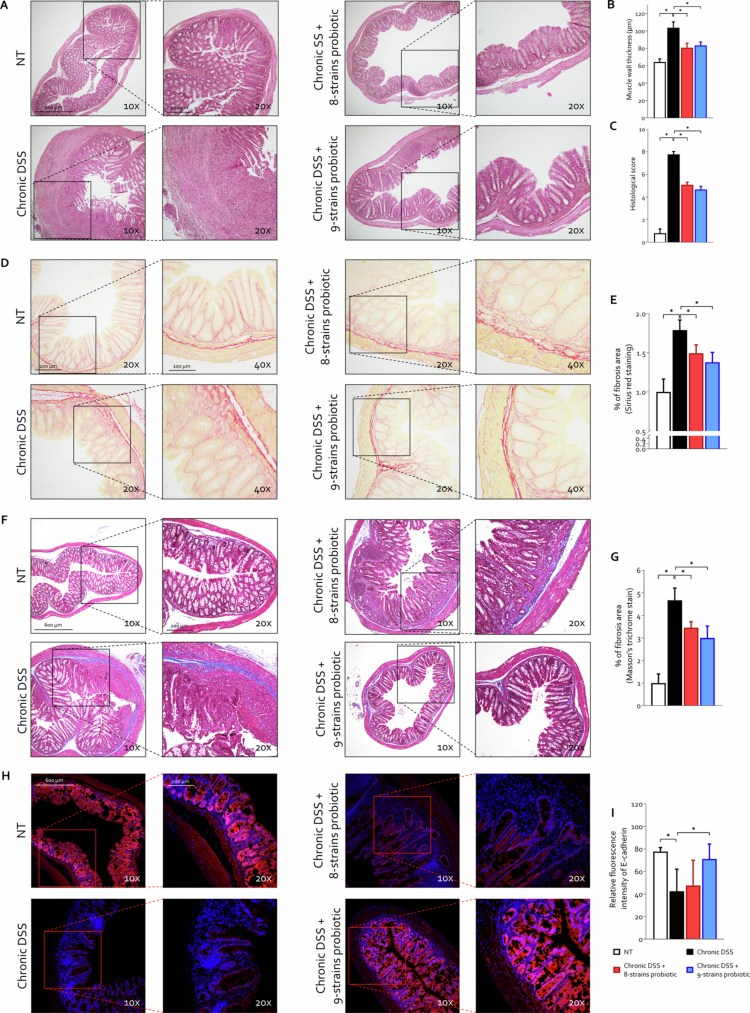
Histopathological evaluation of colonic architecture and epithelial integrity in chronic colitis C57BL/6 male mice were treated with three cycles of DSS alone or in combination with 8-strains probiotic or 9-strains probiotic (50 × 10^9^ bacteria/kg/day). (A) Representative hematoxylin and eosin (H&E)-stained sections of the colon (original magnification 10× and 20×) and (B) measurement of muscularis thickness and (C) histological scoring of tissue damage. (D) Sirius red staining of colon sections (original magnification 20× and 40×) and (E) % of the fibrosis area was normalized to NT experimental group, measured by quantization of Sirius red staining as described in Material and Methods section by Image J analysis. (F) Masson's trichrome staining of colon sections (original magnification 10× and 20×) and (G) % fibrosis area, normalized to the NT experimental group, were measured by quantization of Masson's trichrome staining as described in Material and Methods section. (H) Immunofluorescence staining of colonic sections using an anti-E-cadherin antibody (red) and DAPI (blue) to label nuclei and (I) quantification of E-cadherin fluorescence intensity as described in Material and Methods section. Graphs show mean ± SEM of 5 NT, 10 DSS, 8 DSS+ 8-strains probiotic, and 10 DSS+ 9-strains probiotic mice. Statistical analysis was performed using the Kolmogorov–Smirnov test for normality followed by one-way ANOVA (*p < 0.05).

Subsequently, we performed a detailed histological analysis of the colon (Figure 3). Our data showed that chronic administration of DSS-induced profound structural alterations in the colon, characterized by increased muscular wall thickness, marked elevation of histological scores, and increased collagen deposition, as demonstrated by both Sirius red staining and Masson's trichrome staining (Figure 3A‒G). Moreover, immunofluorescence analysis using anti-E-cadherin antibody revealed reduced expression of this tight junction protein in DSS-treated mice compared to untreated controls, indicating compromised intestinal barrier integrity (Figure 3H, I). Daily treatment with both probiotic formulations significantly mitigated tissue damage, preserving colon structure, reducing collagen deposition, and thus effectively preventing fibrosis development (Figure 3A–G). Notably, the novel 9-strains probiotic formulation exhibited slightly superior efficacy compared to 8-strains probiotic, significantly preserving E-cadherin expression and thereby decreasing intestinal permeability relative to that of DSS-only treated mice (Figure 3H, I).

**Figure 4.**
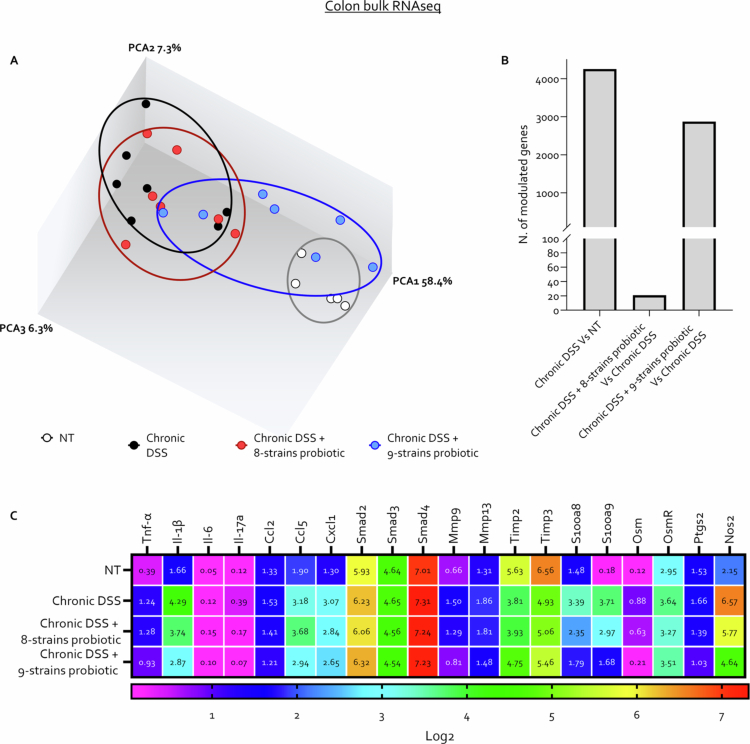
The 9-strains probiotic induces a broader and more profound transcriptomic reprogramming compared to the 8-strains formulation. C57BL/6 male mice were treated with three cycles of DSS alone or in combination with 8-strains probiotic or 9-strains probiotic (50 × 10^9^ bacteria/kg/day). RNA-seq analysis was carried out in colon samples from each experimental group: RNA-seq analysis was performed on colonic samples from each experimental group. (A) Principal coordinate analysis (PCoA) plot of β-diversity illustrating the distribution of samples across groups (each dot represents a sample). (B) Number of differentially expressed genes in chronic DSS-treated mice compared to NT controls and in the DSS+ 8-strains probiotic or DSS+ 9-strains probiotic groups compared to DSS alone group (fold change <−2 or >2, *p value < 0.05). (C) Heatmap showing the expression levels of selected genes involved in inflammatory and fibrotic processes across the experimental groups.

To further elucidate the mechanism of action of the two probiotic formulations, we performed RNA sequencing (RNAseq) analysis on colon tissues from mice across the different experimental groups (Figure 4A‒C). Our data demonstrated that the two formulations had markedly different effects on gene expression profiles (Figure 4A, B). The novel 9-strains probiotic modulated the expression of over 2,500 genes compared to the DSS-only treated group, shifting the gene expression profile closer to that observed in naïve mice (Figure 4A, B). Analysis of selected key genes involved in intestinal inflammation and fibrosis revealed that 9-strains probiotic exhibited greater modulation of gene expression compared to 8-strains probiotic, significantly reducing the expression of proinflammatory cytokines, including *Tnf-α*, *Il-1β*, *Il-6*, and *Il-17a*, as well as chemokines, such as Ccl2, Ccl5, and Cxcl1. Additionally, 9-strains probiotic markedly downregulated the S100a8 and S100a9 genes encoding calprotectin, a known marker of intestinal inflammation, and reduced the expression of *Oncostatin M* (*Osm*), which is implicated in resistance to anti-TNFα therapy (Figure 4C).

**Figure 5.**
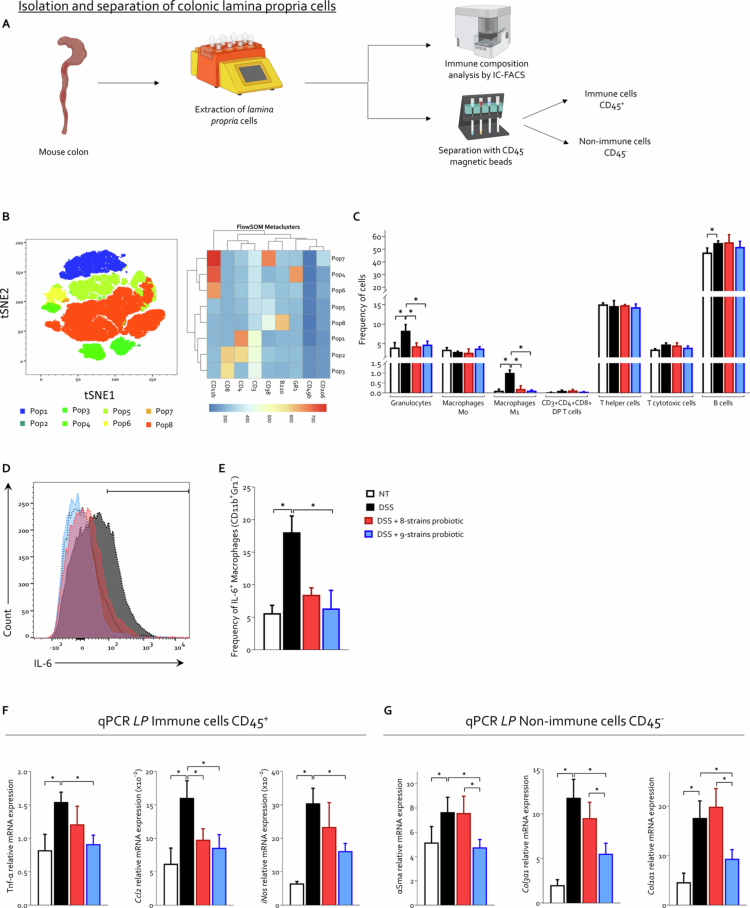
Flow cytometric and qPCR analysis of immune and stromal cells in the colonic *lamina propria* C57BL/6 male mice were treated with three cycles of DSS alone or in combination with 8-strains probiotic or 9-strains probiotic (50 × 10^9^ bacteria/kg/day). At the end of the experiment, the colons were collected, and *lamina propria* cells were isolated. A portion of these cells was used for immune profiling by flow cytometry (IC-FACS), while the remaining cells were sorted using anti-CD45 magnetic beads to separate immune (CD45⁺) and nonimmune (CD45⁻) populations for gene expression analysis of specific markers. (A) Workflow diagram illustrating the protocol for *lamina propria* cell isolation and analysis. (B‒C) To characterize immune cells, a t-distributed stochastic neighbor embedding (tSNE) analysis was performed on CD45⁺ leukocytes, identifying eight distinct subpopulations. (B) Two-dimensional (2D) tSNE plot of leukocyte distribution. (C) Relative frequencies of leukocyte subpopulations across the experimental groups. (D‒E) Frequency of IL-6⁺ macrophages (CD11b⁺GR1⁻) in the different treatment groups. (F) Relative mRNA expression on CD45^+^ cells purified from colonic *lamina propria* cells of inflammatory markers *Tnf-α*, *Ccl2*, and *iNos*. (G) Relative mRNA expression on CD45^-^ cells purified from colonic *lamina propria* cells of fibrosis markers *αSma*, *Col3a1*, and *Col1a1*. The qPCR data were normalized to the mean *Tbp*, *Hprt*, and *Polr2a* mRNA expression. Graphs show mean ± SEM of 5 NT, 10 DSS, 8 DSS+ 8-strains probiotic, and 10 DSS+ 9-strains probiotic mice. Statistical analysis was performed using the Kolmogorov–Smirnov test for normality followed by one-way ANOVA (*p < 0.05).

To further dissect these effects at the cellular level, we isolated cells from the colonic *lamina propria* of mice (Figure 5A). A subset of these cells was utilized for immune cell population identification via flow cytometry (Figure 5B‒E), while the remaining cells were separated into immune (CD45^+^) and nonimmune (CD45^−^, including fibroblasts) fractions for subsequent qPCR analysis (Figure 5F, G).

t-SNE analysis of CD45⁺ leukocytes enabled the characterization of immune cell populations derived from the colonic *lamina propria*. Using a panel of markers, including CD11b, GR1, CD38, CD206, CD49b, CD3, CD8, and B220, we performed unsupervised clustering via FlowSOM metaclusters, which identified eight distinct populations: Pop1 T helper cells (CD11b⁻GR1⁻CD38⁻CD206⁻CD49b⁻CD3⁺CD4⁺CD8⁻B220⁻), Pop2 double positive (DP) T cells (CD11b⁻GR1⁻CD38⁻CD206⁻CD49b⁻CD3⁺CD4⁺CD8⁺B220⁻), Pop3 cytotoxic T lymphocytes (CD11b⁻GR1⁻CD38⁻CD206⁻CD49b⁻CD3⁺CD4⁻CD8⁺B220⁻), Pop4 granulocytes (CD11b⁺GR1⁺CD38⁻CD206⁻CD49b⁻CD3⁻CD4⁻CD8⁻B220⁻), Pop6 M0 macrophages (CD11b⁺GR1⁻CD38⁻CD206⁻CD49b⁻CD3⁻CD4⁻CD8⁻B220⁻), Pop7 M1 macrophages (CD11b⁺GR1⁻CD38⁺CD206⁻CD49b⁻CD3⁻CD4⁻CD8⁻B220⁻), and Pop8 B lymphocytes (CD11b⁻GR1⁻CD38⁺CD206⁻CD49b⁻CD3⁻CD4⁻CD8⁻B220⁺) (Figure 5B). The analysis also identified a population named pop5, composed of ungated cells negative for all tested markers (CD11b⁻GR1⁻CD38⁻CD206⁻CD49b⁻CD3⁻CD4⁻CD8⁻B220⁻), representing approximately 30% of the cells in each sample.

The analysis of population frequencies across experimental groups revealed that DSS-induced colitis, in agreement with hematological data (Figure 2F), promoted an increase in the number of granulocytes and M1 macrophages, along with elevated B lymphocyte counts (Figure 5C). A trend toward increased cytotoxic T cells was also noted, although it did not reach statistical significance. Treatment with either probiotic formulation significantly reduced the percentages of granulocytes and M1 macrophages infiltrating the *lamina propria*, restoring an immune landscape closely resembling that of naïve mice (Figure 5C).

Given the probiotic-mediated modulation of M1 macrophage frequency and considering that the colonic *lamina propria* is a major anatomical site for macrophage accumulation, we further investigated the effects of probiotic treatment within this specific compartment by assessing IL-6 production (Figure 5D, E and Figure S1). Our results showed that chronic DSS exposure increased the frequency of IL-6⁺ macrophages by approximately four-fold compared to naïve controls (Figure 5E), but only *in vivo* administration of 9-strains probiotic significantly reversed this effect, demonstrating a greater immunomodulatory capacity compared to the 8-strains probiotic (Figure 5E).

Gene expression analysis through qPCR on CD45⁺ and CD45⁻ cells isolated from the colonic *lamina propria* revealed that chronic DSS administration induced a marked upregulation of *Tnf-α*, *Ccl2*, and *iNos* in immune cells, which are proinflammatory markers typically associated with macrophage-mediated responses (Figure 5F). On the other hand, in CD45⁻ cells, which include intestinal fibroblasts, the data indicated the activation of fibrotic pathways (Figure 5G). Consistent with the flow cytometric findings, showing modulation of IL-6⁺ macrophage frequency, qPCR analysis of CD45⁺ cells demonstrated that while the 8-strains probiotic exerted only a modest anti-inflammatory effect, the 9-strains probiotic exhibited a robust immunomodulatory activity by significantly downregulating the expression of all the analyzed genes (Figure 5F). This divergence in the efficacy of the two formulations was even more pronounced in CD45⁻ cells: the 8-strains probiotic had no significant impact on fibrotic gene expression, whereas the 9-strains probiotic markedly downregulated all three profibrotic markers investigated (Figure 5G).

### Probiotic-induced restoration of the fecal bile acid pool and reversal of colitis-associated bile acid dysmetabolism

**Figure 6.**
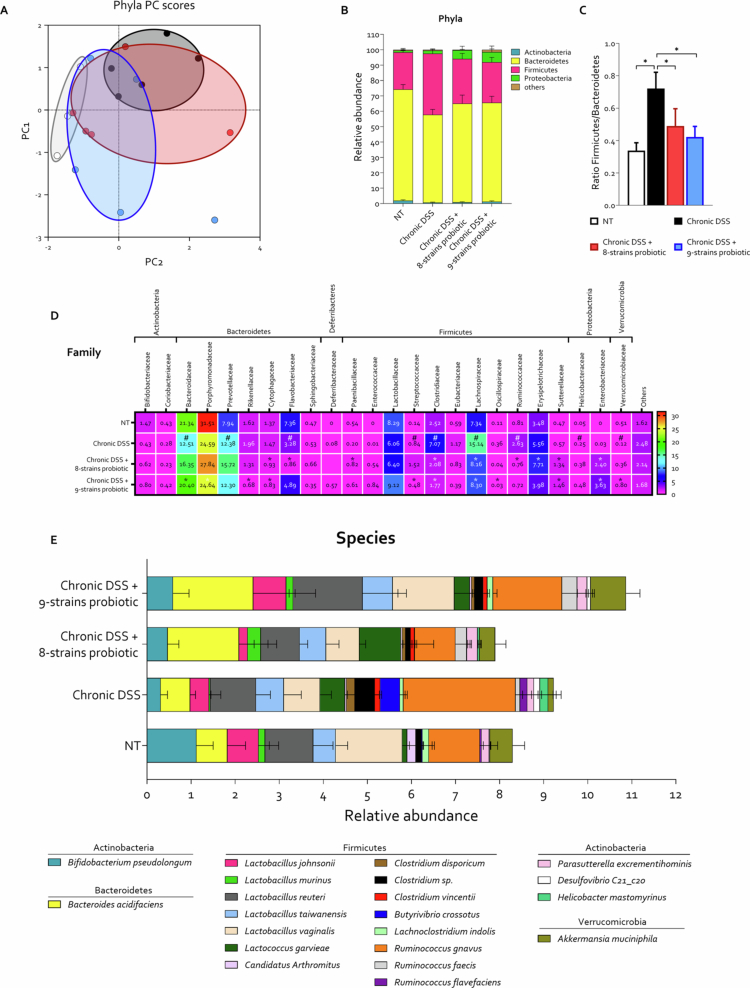
Probiotic-induced modulation of the gut microbiota composition. C57BL/6 male mice were treated with three cycles of DSS alone or in combination with 8-strains probiotic or 9-strains probiotic (50 × 10^9^ bacteria/kg/day). At the end of the experimental protocol, fecal samples were collected from the colon of each mouse for gut microbiota analysis. (A) Taxonomic profiling of the microbiota at the phylum level represented by a principal coordinate analysis (PCoA) plot of β diversity, illustrating the distribution of individual samples (each dot represents a sample). (B) Relative abundance of bacterial phyla in fecal samples. (C) Firmicutes-to-Bacteroidetes relative abundance ratio. (D) Heatmap showing the relative abundance of bacterial families across experimental groups. (E) Bar plot illustrating the relative abundance of bacterial species identified in all experimental samples. Graphs show mean ± SEM of 4 NT, 5 DSS, 5 DSS+ 8-strains probiotic, and 5 DSS+ 9-strains probiotic mice. Statistical analysis was performed using the Kolmogorov–Smirnov test for normality followed by one-way ANOVA (*p < 0.05).

Given the well-established role of gut dysbiosis in the pathogenesis of IBD, we performed a comprehensive analysis of the intestinal microbiota across experimental groups (Figure 6). In the chronic colitis murine model, disease induction resulted in a pronounced dysbiotic shift, characterized by a significant increase in Firmicutes and a concomitant reduction in Bacteroidetes, leading to an elevated Firmicutes/Bacteroidetes ratio, a recognized indicator of disease severity (Figure 6A‒C).^43^ Substantial alterations were also observed at the family level (Figure 6D).

Both probiotic formulations exerted beneficial effects on the microbial community composition, restoring the relative abundances of major phyla and families, and significantly reducing the Firmicutes/Bacteroidetes ratio (Figure 6A‒D). A deeper analysis of the gut microbiota at the species level identified a total of 97 bacterial species (Table S1). We focused our investigation on those species that were detectable in either the untreated mice or in the group with DSS-induced colitis at a relative abundance exceeding 0.1%. This cutoff threshold reduced the number of species considered to be 21, which are displayed in Figure 6E. Microbial community profiling revealed a profound species-level reconfiguration of the gut ecosystem in response to chronic DSS exposure. Chronic DSS administration led to a marked reduction in the abundance of *Bifidobacterium pseudolongum* (*B. pseudolongum*), *Lactobacillus johnsonii* (*L. johnsonii*), *Lactobacillus murinus* (*L. murinus*), *Lactobacillus vaginalis* (*L. vaginalis*), and *Akkermansia muciniphila* (*A. muciniphila*). In contrast, microbiota profiling of mice treated with DSS alone revealed an expansion of *Lactococcus garvieae* (*L. garavieae*), various *Clostridium* species, *Ruminococcus gnavus* (*R. gnavus*), and the emergence of *Butyrivibrio crossotus*. Supplementation with the 8-strains probiotic significantly attenuated DSS-induced shifts. Relative abundances of *B. pseudolongum* and *L. johnsonii* restored to near-basal levels. *Lactobacillus johnsonii* has recently been shown to rebalance Treg/Th17 responses and downregulate colonic TNF-α, IL-1β, and IL-6 in DSS colitis.^44^

The overrepresentation of *R. gnavus* and *Clostridium* spp. was curtailed, while *A. muciniphila* increased by ~6-fold relative to that of DSS alone, indicating partial restoration of barrier homeostasis.^45^ The next-generation probiotic consortium 9-strains probiotic exerted the most pronounced ecological impact. The data shown an enrichment of additional beneficial taxa, such as *Bacteroides acidifaciens*, showed to synthesize the anti-inflammatory fatty acid pentadecanoic acid and protect against DSS colitis^46^ and *L. vaginalis*, which alleviates colitis through AhR-active indoleacrylic acid production.^47^ On the other hand, reduction of pro-colitogenic *Clostridium spp*., favoring re-establishment of a butyrate-rich, Treg-inducing community; marked expansion of *A. muciniphila* and replenishment of SCFA-producing *B. pseudolongum*,^48^^,^^49^ driving the community toward a configuration closely resembling the NT profile. Chronic DSS elicits a dysbiotic fingerprint typified by enrichment of proinflammatory taxa and depletion of key symbionts. Both probiotic formulations counteracted this dysbiosis, albeit with markedly different effects on the composition of the gut microbiota.

Species-level analysis revealed that none of the bacterial strains included in the two probiotic formulations were detectable in the fecal microbiota of mice treated with these probiotics, suggesting that the beneficial effects observed may not be due to stable intestinal colonization but rather to the metabolic activity of the administered bacteria.

**Figure 7.**
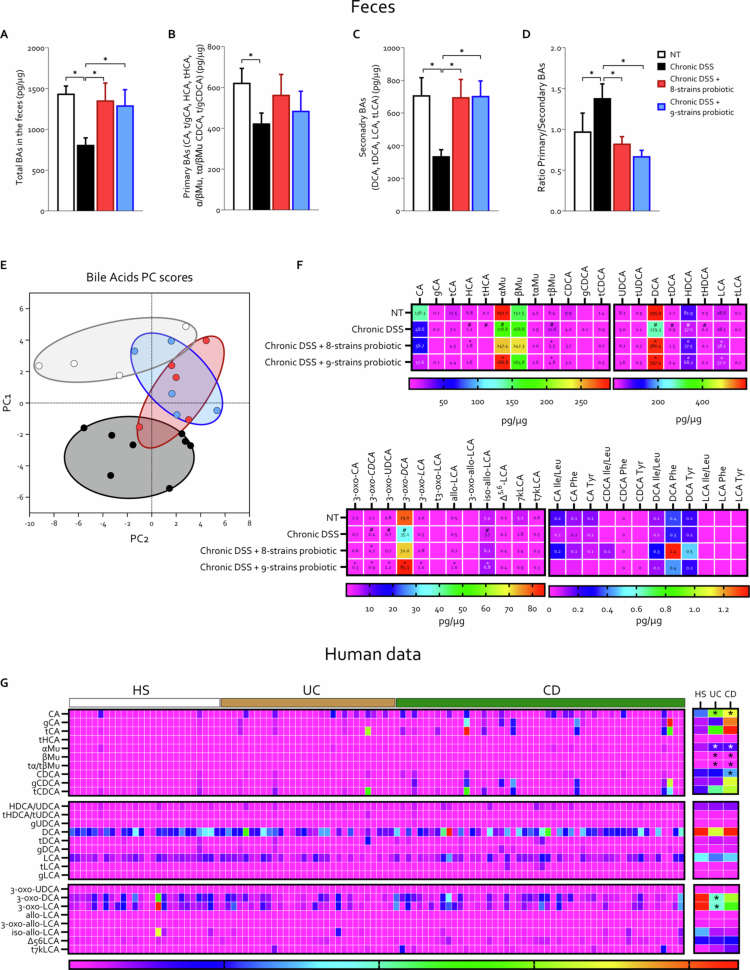
Fecal bile acid profiling reveals metabolic differences between probiotic formulations. C57BL/6 male mice were treated with three cycles of DSS alone or in combination with 8-strains probiotic or 9-strains probiotic (50 × 10^9^ bacteria/kg/day). Analysis of fecal bile acids: (A) total fecal bile acid pool (pg/μg), (B) concentration of primary bile acids (pg/μg), (C) concentration of secondary bile acids (pg/μg), and (D) ratio of primary to secondary bile acids. (E) Principal coordinate analysis (PCoA) plot of β diversity based on fecal bile acid profiles showing the distribution of individual samples (each dot represents a sample) and (F) individual concentrations of bile acids and their derivatives in feces (pg/μg). Graphs show mean ± SEM of 4 NT, 8 DSS, 5 DSS+ 8-strains probiotic, and 5 DSS+ 9-strains probiotic mice. (G) We utilized the IBD cohort from the Human Microbiome Project (HMP2) to investigate the fecal bile acid content in healthy individuals, patients with UC, and patients with CD. The heatmap on the left displays the fecal concentrations of 27 distinct bile acid species in individual samples from healthy subjects, UC patients, and CD patients. The heatmap on the right shows the average fecal concentration of each bile acid species across the three groups: healthy subjects (HSs), ulcerative colitis (UC), and Crohn's disease (CD) patients. Statistical analysis was performed using the Kolmogorov–Smirnov test for normality followed by one-way ANOVA (*p < 0.05).

Among the numerous metabolites produced by the gut microbiota, bile acids have emerged as key mediators of host–microbiota interactions and are increasingly recognized as major microbial-derived metabolites of growing interest in biomedical research.^14^^,^^50^^,^^51^ Given the emerging evidence supporting a central role for gut microbiota-derived metabolites, particularly secondary bile acids, in the pathogenesis of IBD, we next analyzed the fecal bile acid profile across the experimental groups (Figure 7 and Figure S2). Our data showed that chronic DSS-induced colitis profoundly disrupted the fecal bile acid pool, resulting in a marked reduction in the total bile acid content. Notably, this decrease was primarily driven by a significant loss of secondary bile acids, leading to a substantial increase in the ratio of primary to secondary bile acids (Figure 7A–D). Quantification of 44 distinct bile acid species in the fecal samples revealed a substantial shift in the bile acid profile of the DSS-treated mice compared to naïve controls (Figure 7E, F and Figure S2). Specifically, chronic DSS exposure led to a significant decrease in several bile acids, including hyodeoxycholic acid (HCA), α-muricholic acid (αMu), taurine-conjugated β-muricholic acid (tβMu), deoxycholic acid (DCA), taurodeoxycholic acid (tDCA), hyodeoxycholic acid (HDCA), taurohyodeoxycholic acid (tHDCA), 3-oxo-CDCA, 3-oxo-UDCA, 3-oxo-DCA, and iso-allo-LCA (Figure 7F). These findings highlight a pronounced reduction in microbiota-derived secondary bile acids in colitic mice. This pattern mirrors observations in IBD patients, who exhibit a marked decrease in fecal levels, especially 3-oxo-DCA and 3-oxo-LCA (Figure 7G).

Administration of both probiotic formulations increased the total fecal bile acid pool and significantly restored the concentration of secondary bile acids, thereby reducing the primary-to-secondary bile acid ratio (Figure 7A–D). Principal component analysis (PCA) of the bile acid profiles confirmed that probiotic treatment shifted the fecal bilome toward that of naïve mice, while the DSS-only group clustered distinctly apart (Figure 7E). Notably, the novel 9-strains probiotic formulation exerted a stronger effect, as evidenced by two samples clustering closely with the naïve group and none overlapping with the DSS group (Figure 7E).

Detailed analysis of the 44 bile acid species profiled in fecal samples revealed that the two probiotic formulations exerted markedly different effects on bile acid modulation. The 8-strains probiotic significantly altered the concentration of only six bile acids (*HCA*, *tbMU*, *DCA*, *HDCA*, *LCA*, and *3-oxo-CDCA*). In contrast, the 9-strains probiotic significantly increased the fecal levels of *αMu*, *tβMu*, *DCA*, *HDCA*, *LCA*, *3-oxo-CA*, *3-oxo-CDCA*, *3-oxo-UDCA*, *3-oxo-DCA*, *3-oxo-LCA*, *allo-LCA*, and *iso-allo-LCA*, thereby restoring a broader spectrum of microbiota-derived bile acid metabolites (Figure 7F).

Bile acids are well known not only for their ability to emulsify dietary lipids and facilitate their intestinal absorption but also for acting as endogenous ligands for a specific class of receptors collectively referred to as bile acid-activated receptors (BARs).^11^^,^^52^ Within this class, the two most extensively studied and characterized receptors are FXR, a nuclear receptor, and GPBAR1, a G protein-coupled membrane receptor, which are primarily activated by primary and secondary bile acids, respectively.^11^ These two receptors are abundantly expressed in epithelial cells, neurons, glial cells, endocrine cells, including pancreatic β-cells and L cells, as well as in extracellular matrix (ECM) cells and immune cells.^53^^,^^54^ In immune cells, activation of FXR and GPBAR1 by their respective ligands triggers a counter-regulatory program that supports the maintenance of a tolerogenic phenotype within the intestinal and hepatic immune systems.^11,55‐58^

**Figure 8.**
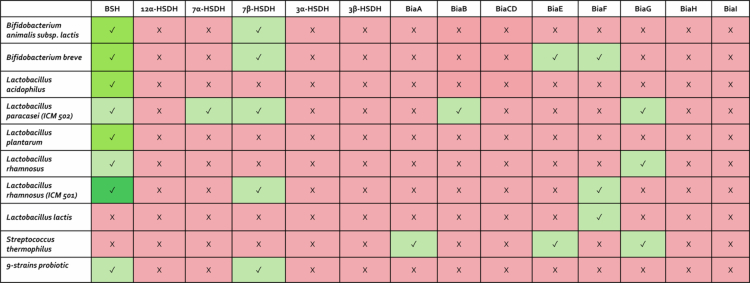
Proteomic evidence of bile acid–transforming enzymes in the 9-strains probiotic. Proteomic analysis of bile acids synthesis-involved proteins across the bacterial strains and the 9-strains probiotic. Identified proteins are depicted in green, according to Proteome Discoverer coverage values (light green <30%, green <70%, and dark green >70%); the unidentified proteins are depicted in red.

**Figure 9.**
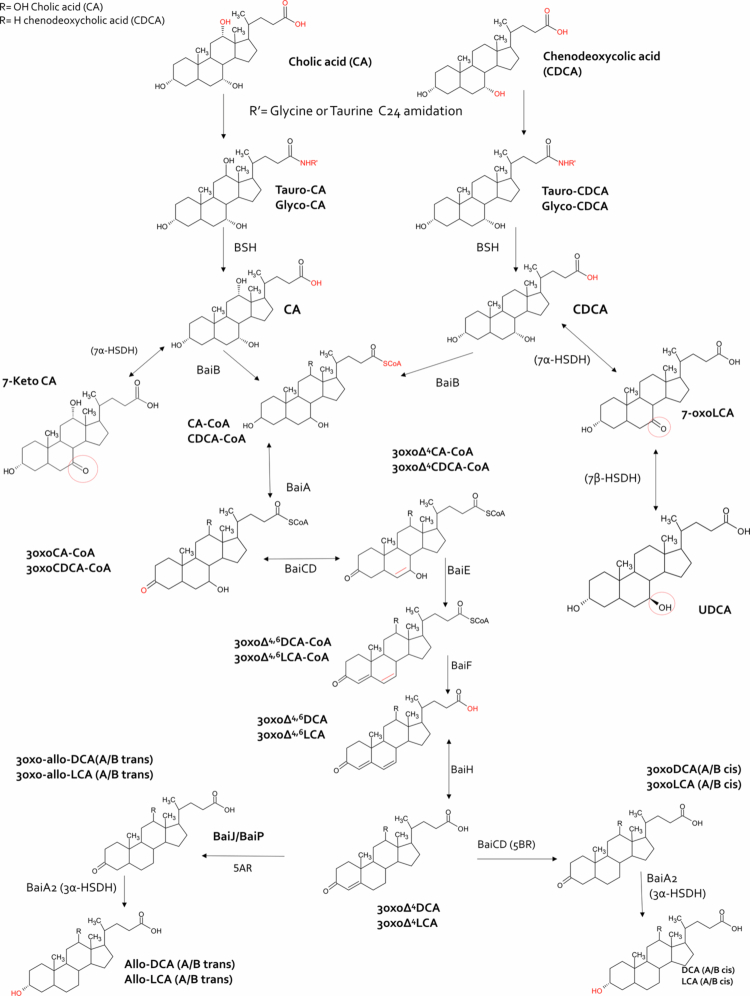
Bile acid biosynthetic pathway. Schematic representation of the biosynthesis of secondary bile acids and their derivatives from free and conjugated primary bile acids.

To investigate the potential production of secondary bile acids and their derivatives by 9-strains probiotic, we performed a proteomic analysis to assess the presence of enzymes involved in secondary bile acid biosynthesis within each individual strain and in the complete 9-strains probiotic formulation (Figure 8 and Table S2). The data demonstrated that, among the proteins involved in bile acid biosynthesis, most of the bacterial strains expressed bile salt hydrolase (BSH) and 7β-hydroxysteroid dehydrogenase (7β-HSDH) (Figure 8), which catalyze the initial transformations of primary bile acids, ultimately leading to the formation of secondary bile acids (lithocholic acid, LCA, and deoxycholic acid, DCA) and their microbial derivatives (Figure 9).

**Figure 10.**
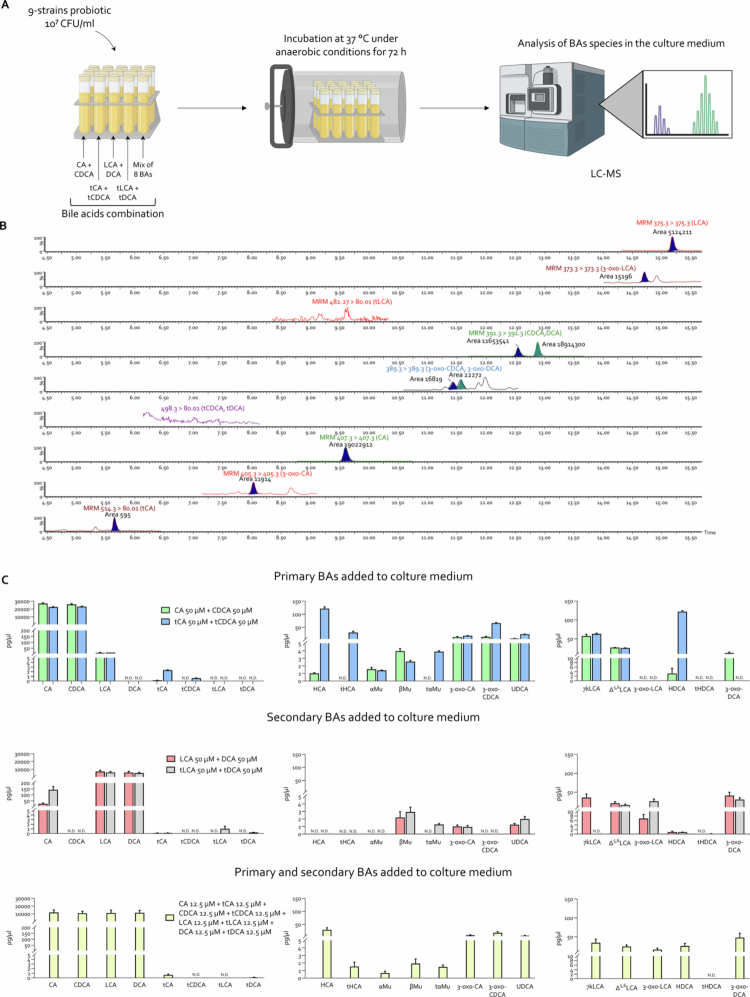
*In vitro* study of bile acid metabolism by the 9-strains probiotic. (A) Workflow diagram illustrating the culture protocol for the 9-strains probiotic in the presence of bile acids. (B) An example of an LCMS trace of the methanol extract of the bacterial mixture 9-strains probiotic supernatant, to which primary, secondary, and conjugated BAs have been added in culture broth. Along with the specifics of the multiple reaction monitoring (MRM) experiment and the associated peak area, each chromatographic trace displays the peaks of the related bile acids as conjugated, unconjugated, primary, secondary, and their oxidized congeners at position 3. (C) Individual concentrations of bile acids and their derivatives in bacterial culture medium (pg/μL). Graphs show mean ± SEM of three samples for experimental groups. N.D. = not detectable.

To further elucidate the involvement of probiotics in bile acid metabolism, we first carried out a proteomic analysis of bile acid-metabolizing enzymes in the 9 strains of bacteria used in this study, and in addition, we have cultured the 9-strains mixture under anaerobic conditions for 72 h in the presence of different combinations of bile acids (see the dedicated section in Materials and Methods). Metabolites were subsequently quantified in the culture medium via LC‒MS (Figure 10 and Figure S3). The proteomic analysis (Figure 8), demonstrated that all strains of probiotics express the BSH, the key deconjugating enzyme, and 9-strains mixture efficiently deconjugates the tauro-conjugated bile acids. Thus, as shown in Figure 10, primary or secondary tauro-conjugated bile acids were efficiently converted into the corresponding unconjugated primary and secondary bile acids, confirming the presence of functional BSH(s). These results were consistent with the observation that *in vivo* treatment with the 9-strains formulation shift fecal bile acid composition toward secondary bile acids was observed. These *in vivo* metabolomic studies also demonstrated that the 9-strains probiotic formulation efficiently process LCA and DCA toward their 3- and 7-oxo derivatives with the generation of 3-oxo-DCA (Figure 10), mirroring the *in vivo* results (Figure 7F).

**Figure 11.**
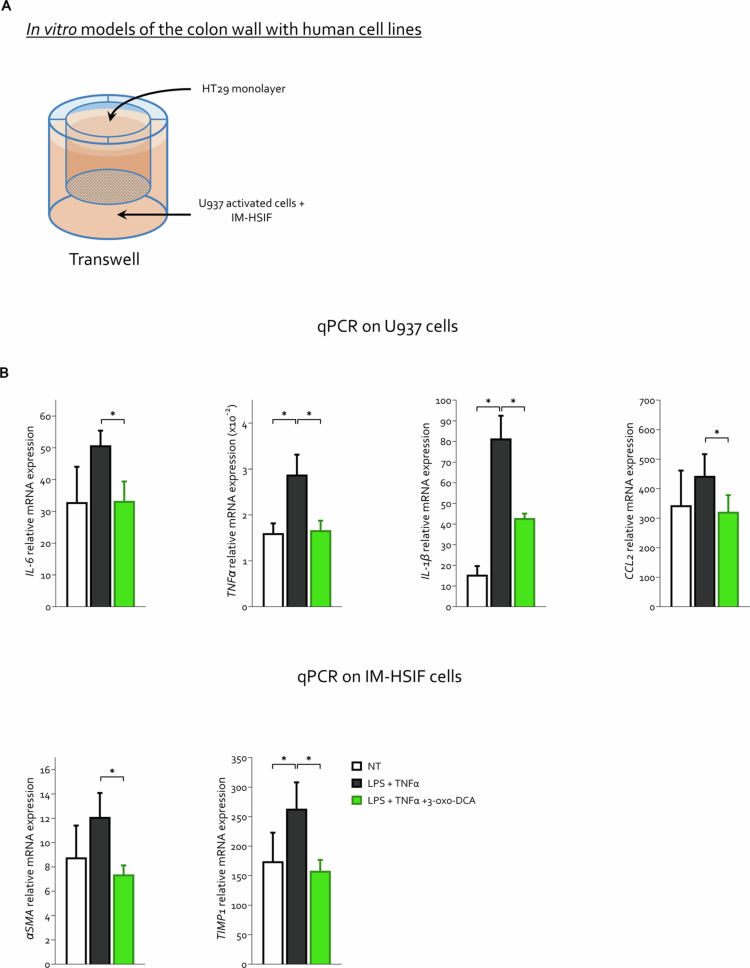
3-oxo-DCA replicates the anti-inflammatory and antifibrotic effects of the 9-strains probiotic in a human intestinal coculture model. (A) A coculture system was established by seeding HT29 (epithelial adenocarcinoma) cells as a confluent monolayer on the upper insert of Transwell® chambers, while a combination of predifferentiated U937 (human monocyte)-derived macrophages and IM-HSIF (human small intestinal fibroblasts) was placed in the lower compartment for *in vitro* reconstruction of the intestinal niche. Cells were stimulated for 72 h with a combination of LPS and TNF-α, which simulate dysbiosis and inflammation, either alone or in the presence of 3-oxo-DCA. Additional details on the coculture protocol are provided in the corresponding paragraph of the Materials and Methods section. (B, C) Relative mRNA expression of (C) cytokines and chemokines and (D) fibrotic markers measured in U937 + IM-HSIF cells. The qPCR data were normalized to the mean *HPRT* and *POLR2A* mRNA expression. Graphs show mean ± SEM of five samples for experimental groups. Statistical analysis was performed using the Kolmogorov–Smirnov test for normality followed by one-way ANOVA (*p < 0.05).

Among the bile acids modulated by 9-strains probiotic administration, the most markedly increased was 3-oxo-DCA, a potent ligand of GPBAR1, whose concentration increased by 2.4-fold compared to fecal levels measured in DSS-treated mice (Figure 7F) and, whose production by the probiotic formulation was also demonstrated *in vitro* (Figure 10). We therefore investigated whether 3-oxo-DCA could replicate the anti-inflammatory and antifibrotic effects exerted by 9-strains probiotic in the chronic colitis mouse model. To this end, we established an *in vitro* coculture system consisting of HT29 cells seeded on the upper side of a Transwell® insert to form an epithelial monolayer and human intestinal fibroblasts together with predifferentiated U937-derived macrophages in the lower compartment, thus mimicking the intestinal niche (Figure 11A). A detailed description of the experimental setup is provided in the Materials and Methods section.

The cocultures were stimulated for 72 h with LPS and TNF-α, either alone or in combination with 3-oxo-DCA. At the end of the stimulation period, U937 and IM-HSIF cells were separated by magnetic cell sorting with CD45 MicroBeads and used for gene expression analysis via quantitative PCR (Figure 11B, C).

In U937 and IM-HSIF cells, LPS + TNF-α stimulation resulted in robust induction of proinflammatory cytokines typically associated with M1 macrophage activation, recapitulating the immune profile observed *in vivo* in DSS-treated mice (Figure 5) and of genes that suggest a profibrotic phenotype in fibroblasts (Figure 11B, C). Notably, co-treatment with 3-oxo-DCA significantly attenuated the expression of proinflammatory cytokines in the U937 cell line and the chemokine CCL2 and inhibited the transition of fibroblasts toward a profibrotic phenotype.

### GPBAR1 mediates the anti-inflammatory and antifibrotic effects exerted by probiotic administration in chronic colitis

**Figure 12.**
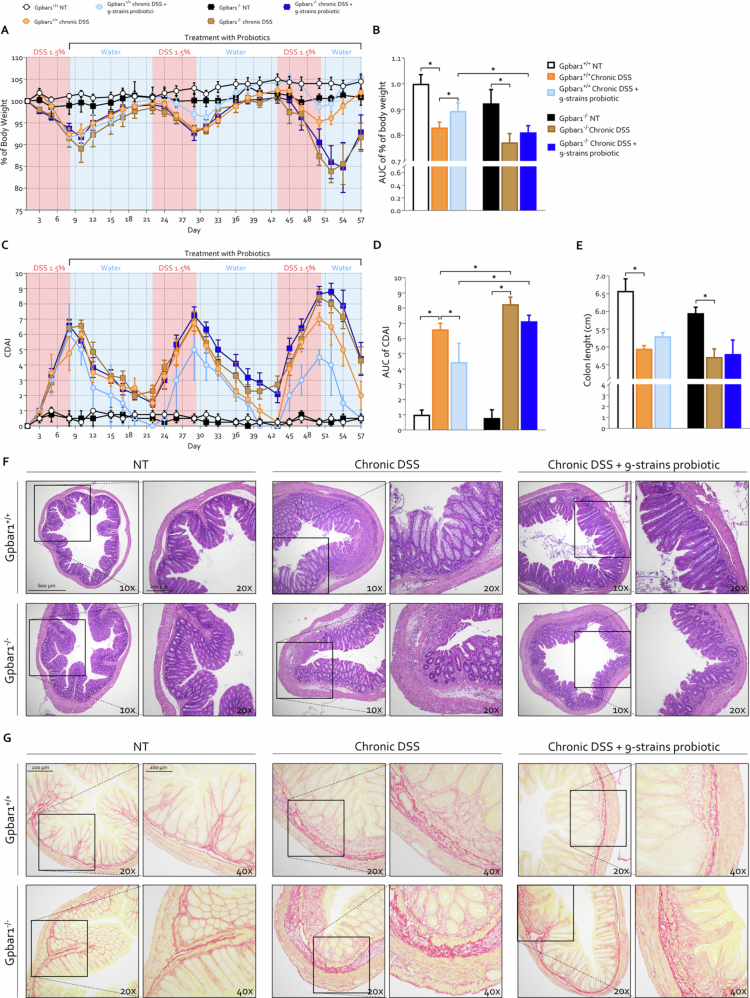
GPBAR1 mediates the therapeutic effects of the 9-strains probiotic in chronic colitis Gpbar1^+/+^ and Gpbar1^⁻/⁻^ male mice were treated with three cycles of DSS, either alone or in combination with 8-strains probiotic neo9 (5 × 10¹⁰ bacteria/kg/day). (A) Changes in body weight and (B) corresponding AUC normalized to that of Gpbar1^+/+^ NT experimental group. (C) Colitis Disease Activity Index (CDAI) and (D) AUC normalized to the Gpbar1^+/+^ NT experimental group. (E) Colon length (cm). (F) Representative hematoxylin and eosin (H&E)-stained colon sections (original magnification 10× and 20×). (G) Representative Sirius red-stained colon sections used to assess collagen deposition (original magnification 20× and 40×). Graphs show mean ± SEM of 5 Gpbar1^+/+^ NT, 7 Gpbar1^+/+^ DSS, 7 Gpbar1^+/+^ DSS+ 9-strains probiotic, 5 Gpbar1^⁻/⁻^ NT, 7 Gpbar1^⁻/⁻^ DSS, 7 Gpbar1^⁻/⁻^ DSS+ 9-strains probiotic mice. Statistical analysis was performed using the Kolmogorov–Smirnov test for normality followed by one-way ANOVA (*p < 0.05).

The majority of bile acids found to be increased in the feces of mice treated with 9-strains probiotic are potent agonists of GPBAR1,^17^^,^^20^ suggesting that the beneficial effects of the probiotic formulation may be, at least in part, mediated by this receptor. GPBAR1 is the main receptor for secondary bile acids and their microbial derivatives, a key regulator of intestinal inflammation, epithelial barrier function, immune homeostasis, and fibrotic remodeling.^20^^,^^59^ To determine whether the beneficial effects exerted by 9-strains probiotic were mediated by the production of secondary bile acids and subsequent activation of GPBAR1, we conducted a chronic DSS-induced colitis protocol in both Gpbar1^+/+^ and Gpbar1^−/−^ mice treated with 9-strains probiotic (Figure 12).

Gpbar1 ablation rendered mice more susceptible to developing severe disease progression over time, with pronounced differences especially evident following the final DSS administration cycle, as demonstrated by increased weight loss and a significantly higher CDAI compared to their *Gpbar1*^*+/+*^ counterparts (Figure 12A‒D). Administration of the probiotic formulation 9-strains probiotic reversed disease symptoms in wild-type mice, which was consistent with findings from the previous experiment (Figure 2), but exerted only a modest beneficial effect in *Gpbar1*^-^^*/*^^-^ mice. Macroscopic and microscopic analyses of colonic tissues confirmed that Gpbar1^−/−^ mice developed more severe colitis upon DSS treatment, as previously reported by our group.^58^ In addition, these mice showed increased intestinal fibrosis, highlighting the involvement of GPBAR1 in fibrotic processes as well (Figure 12E‒G).

Moreover, the beneficial effect of 9-strains probiotic administration on intestinal fibrosis, observed in *Gpbar1*^*+/+*^ mice, was completely abolished in knockout mice. Taken together, these results indicate that the anti-inflammatory and antifibrotic effects exerted by 9-strains probiotic are partially mediated by GPBAR1 activation, strongly suggesting that a substantial component of the probiotic's beneficial activity arises from microbial-derived secondary bile acids and their metabolites, for which GPBAR1 serves as the primary receptor.

## Discussion

Secondary bile acids generated by intestinal microbial enzymes are an essential component of the network of chemical signals that mediates communication between the microbiota and the host. In addition to the so-called MDBA,^60^ generated by BSH-expressing bacteria, modifications of the two main secondary bile acids, LCA and DCA by oxidation, and the dihydroxylation of one or more OH groups carried out by hydroxysteroid dehydrogenases (HSDHs), along with epimerization, generate a cluster of chemically different bile acids, such as 3-, 7-, and 12-oxo-LCA and DCA, allo-LCA and DCA, iso-allo-LCA, UDCA, and hycholic acid (HCA) derivatives.^13^ In contrast to primary bile acids, which function as FXR ligands, these secondary bile acids are preferential ligands for GPBAR1, VDR, and RORγt.^11^

While there is evidence that these secondary bile acids exert beneficial effects in maintaining host immune homeostasis and contribute to microbial stability,^59^ the role they play in mediating probiotic functionality has been poorly investigated. In the present study, we provide evidence that the modulation of the intestinal microbiota by a multistrain probiotic mixture in a mouse model of IBD generates a cluster of secondary bile acids essential for probiotic functionality. By integrating multiomics profiling, immunophenotyping, histopathological assessments, and mechanistic validation in GPBAR1-deficient mice, we demonstrate that these bile acids converge on GPBAR1 and that the receptor is essential for eliciting beneficial effects on probiotics in a mouse model of intestinal inflammation and fibrosis.

Here, we have compared the functionality of two multistrains probiotics formulations in a mouse model of intestinal inflammation/fibrosis. The first formulation is an 8-strains combination of *L. paracasei* subsp. *paracasei*, *L. plantarum*, *L. acidophilus*, *L. delbrueckii* subsp. *bulgaricus*, *B. longum* subsp. *longum*, *B. breve*, *B. longum* subsp. *infantis*, *and S. salivarius* subsp. *thermophilus* commercially available under various brand names (including Vivomixx® used in this study) worldwide. The second is a novel 9-strains formulation of *L. paracasei*, *L. rhamnosus* IMC 501, *L. rhamnosus MC502*, *B. breve*, *B. lactis*, *L. acidophilus*, *L. plantarum*, *Lactococcus lactis*, and *Streptococcus thermophilus* (commercially available under the names Vivomixx neo9®). Both formulations used are available as a blend of high concentrations of living bacterial cells. The two formulations differ in terms of strain and the 9-strains probiotic contains a large amount (approximately 50%) of *L. rhamnosus* that, in contrast, is not included in the classical 8-strains formulation. Although the two formulations are not directly comparable in terms of probiotic composition, the classical 8-strains formulation has been widely investigated in models of IBD and has been approved, though only in UC-pochitis patients, for clinical use. With these limitations in mind, we have carried out a head-to-head comparison of the two formulations in models of intestinal fibrosis that remains the major complication of IBD for which no specific therapy is currently available.^61^

In the acute setting of DSS-induced colitis, both the 8-strains and 9-strains probiotic formulations demonstrated comparable therapeutic efficacy. Treatment with either formulation improved body weight, CDAI scores, histological damage, and leukocyte infiltration in the colonic *lamina propria*. These results suggest that, under conditions of acute epithelial injury and inflammation, both formulations are capable of exerting short-term immunomodulatory and epithelial-protective effects.

However, in the chronic model of colitis, which better recapitulates the long-term pathophysiological features of human IBD, including fibrosis and sustained immune activation, a divergent efficacy profile was identified. In this model, administration of the 8-strains formulation exerted partial protection, marginally reducing CDAI scores and circulating inflammatory cell counts, while failing to significantly impact weight loss, colonic fibrosis, or epithelial barrier disruption. In contrast, the 9-strains formulation exhibited a robust and comprehensive therapeutic effect, robustly attenuating the clinical, histological, and molecular hallmarks of disease. Specifically, treatment with the 9-strains probiotic reduced tissue inflammation and collagen deposition, restored epithelial E-cadherin expression, and markedly improved colon architecture.

These differences were further substantiated at the cellular and molecular levels. By flow cytometry analysis of *lamina propria* infiltrating cells, we found that both formulations reduced the frequency of proinflammatory myeloid populations, such as granulocytes and M1-like macrophages, but only the 9-strains formulation effectively attenuated the expansion of IL-6⁺ macrophages, which are strongly associated with chronic intestinal inflammation and fibrosis development in CD patients^62^^,^^63^ (Figure 5). Complementary qPCR analysis of isolated CD45⁺ immune and CD45⁻ stromal cells confirmed that the 9-strains formulation downregulated the expression of proinflammatory cytokines (*Tnf-α*, *Ccl2*, and *iNos*) and fibrogenic markers (α*-Sma*, *Col3a1*, and *Col1a1*), highlighting its superior effectiveness in modulating both the immune and mesenchymal compartments of the inflamed intestine (Figure 5).

We have shown that these effects were mechanistically linked to microbial metabolism rather than to persistent colonization by probiotic strains. Fecal microbiota analysis showed that none of the bacterial species contained in the administered formulations were stably detectable in the feces of treated mice, suggesting that the therapeutic benefits are primarily mediated by transient metabolic interactions rather than by long-term engraftment. Notably, the 9-strains formulation induced a broader and robust reshaping of the microbiota structure, reversing intestinal dysbiosis and selectively enriching the microbiota via beneficial commensals such as *B. acidifaciens*,^64^^,^^65^ *A. muciniphila*,^45^ and *L. vaginalis*,^47^ while reducing proinflammatory taxa, including *Clostridium* spp. and *R. gnavus*^66^ (Figure 6).

Among the many metabolic pathways influenced by the microbiota, those linked to bile acid metabolism^13^ have emerged as a major differentiating factor between the two formulations (Figure 7). Bile acid profiling revealed that while both formulations partially restored the fecal bile acid pool disrupted by DSS administration, the 9-strains probiotic promoted a robust recovery of secondary bile acids and their oxidized derivatives, including the 3-oxo derivatives (e.g., 3-oxo-DCA, 3-oxo-LCA), allo- and iso-allo-derivatives of LCA, and other known GPBAR1 agonists^11^^,^^17^ (Figure 7). These changes translated into a significant improvement in the primary-to-secondary bile acid ratio, which was pathologically elevated in DSS-treated animals.^67^

Our *in vitro* data further reinforce the concept that the 9-strains probiotic possesses an intrinsic capacity to remodel the bile acid pool through active microbial metabolism (Figures 8–10). Consistent with the proteomic signatures indicating the expression of bile salt hydrolase and 7β-hydroxysteroid dehydrogenase across multiple strains, the consortium efficiently deconjugated tauro-conjugated primary and secondary bile acids and catalyzed their conversion into secondary and oxidized derivatives. These metabolic activities mirrored the bile acid shifts observed *in vivo* (Figure 7), including the production of 3-oxo-DCA and other GPBAR1-active metabolites. These experiments demonstrate that the consortium as a whole operates as a functionally competent metabolic unit capable of generating a diversified panel of bile acids derivatives. This concordance between proteomic evidence, *in vitro* metabolic outputs, and *in vivo* bile acid signatures strengthens the conclusion that the therapeutic effects of the 9-strains formulation are driven by its ability to reestablish a complex and functional bile acid network.

To mechanistically validate the relevance of this metabolic rewiring, we employed both *in vitro* and *in vivo* approaches. In a coculture system mimicking the inflamed intestinal niche, treatment with 3-oxo-DCA alone was sufficient to recapitulate the anti-inflammatory and antifibrotic effects observed with the 9-strains probiotic, reducing both cytokine production and fibroblast activation. Moreover, experiments in GPBAR1-deficient mice revealed that the beneficial effects of the 9-strains probiotic on fibrotic remodeling were abrogated in the absence of the receptor. These findings strongly suggest that GPBAR1 serves as a key molecular effector of the probiotic's therapeutic activity, and that the increased production of GPBAR1-active bile acids by the 9-strains formulation underlies its superior efficacy in chronic colitis.

These findings might have translational implications. Indeed, since 3-oxo-DCA was found to be effective in reversing the activation of intestinal fibroblasts and its administration suffices to reverse intestinal inflammation in models of colitis,^17^^,^^19^ it might be speculated that this agent could recapitulate probiotic effects in clinical settings. However, it should be considered that oxobile acids are potent detergents and might alter the microbiota composition.^68^

One limitation of the present study is that our focus on GPBAR1 might have obscured the beneficial role of other receptors, such as VDR or RORγt.^15^ These receptors have been shown to mediate several immune-regulatory effects of secondary bile acids, and their activation could also support the beneficial effects of probiotics.^11^^,^^18^^,^^69^

Taken together, our data demonstrate that while both probiotic formulations are effective in ameliorating acute inflammation, only the 9-strains probiotic exerts a sustained and multifaceted therapeutic effect in the context of chronic intestinal inflammation and fibrosis. This superior efficacy is not merely a consequence of taxonomic changes in the microbiota but is driven by profound remodeling of microbial metabolism, particularly the restoration of a diversified and functionally active bile acid pool. The ability of the 9-strains formulation to promote the production of GPBAR1-active bile acid derivatives and to engage the GPBAR1 signaling axis represents a critical mechanistic advantage, positioning this formulation as a promising next-generation probiotic strategy for the treatment of IBD. These findings underscore the importance of integrating microbial metabolic profiling into the development and evaluation of probiotic therapies and provide a compelling rationale for targeting the bile acid–GPBAR1 axis as a therapeutic avenue in chronic inflammatory and fibrotic diseases of the gut.

## Supplementary Material

Supplementary_Table_2_Proteomica.xlsx Supplementary_Table_2_Proteomica.x

Figure S2.tif Figure S2.tif

Supplementary_Table_1_Species.xlsx Supplementary_Table_1_Species.x

Figure S1.tif Figure S1.tif

Supplementary Material Figure S3 R1.tif

Supplementary Material Supplementary figures legend.docx

## Data Availability

The complete RNA-seq data of mouse colon and the analysis of mouse intestinal microbiota presented this study are openly available in Mendeley data repository DOI: 10.17632/29wmbphbzc.1.
