# Correction

**DOI:** 10.1080/19490976.2026.2692251

**Published:** 2026-06-22

***Addendum to the conflict of interest***

**Article title:** A bile acid–GPBAR1 network supports anti-inflammatory and anti-fibrotic benefits of probiotics in colitis

**Authors:** Michele Biagiolia, Cristina Di Giorgioa, Silvia Marchianòa, Benedetta Sensinia, Ginevra Urbania, Eleonora Giannellia, Ginevra Lachia, Carmen Massaa, Maria Rosaria Settea, Francesca Paniconia, Elva Morrettab, Maria Chiara Montib, Angela Zampellab, Eleonora Distruttic and Stefano Fiorucci

**Journal:** *Gut Microbes*

**DOI:** https://doi.org/10.1080/19490976.2026.2645125

For the avoidance of doubt, the authors wish to provide the following clarification.

Prof. Stefano Fiorucci, Prof. Angelo Zampella, and Dr. Emanuela Distrutti are co-founders of Precision BioTherapeutics S.r.l., a spin-off company of the University of Perugia established in 2019 with the participation of several entities, including Mendes S.A.

Neither Precision BioTherapeutics S.r.l. nor Mendes S.A. had any role whatsoever in the conception, design, funding, conduct, analysis, interpretation, writing, review, or publication of the present study.

This disclosure was not provided upon submission, as the authors did not consider this a competing interest, however, upon query by the journal, this clarification is provided in the interest of transparency and compliance with the journal editorial policies. The authors are unaware of any evidence demonstrating an actual conflict of interest or any influence on the conduct, reporting, or interpretation of the research.

The journal confirms that this competing interest disclosure would have had no impact on the original editorial handling of the article, nor on the original decision made to accept the article.

