# Supplementary material for: A bile acid–GPBAR1 network supports anti-inflammatory and anti-fibrotic benefits of probiotics in colitis
Source: Gut Microbes. 2026 Mar 18;18(1):2645125. doi: 10.1080/19490976.2026.2645125 (PMC13003854; doi:10.1080/19490976.2026.2645125)
Supplement: Supplementary Material — Supplementary figures legend.docx [file KGMI_A_2645125_SM1386.docx]

**Supplementary Figure legends:**

**Figure S1.** C57BL/6 male mice were treated with 3 cycles of DSS alone or in combination with 8-strains probiotic or 9-strains probiotic (50*10^9 bacteria/kg/day). Gating strategy for flow cytometric analysis of IL-6 expression in colonic *lamina propria* macrophages.

**Figure S2.** C57BL/6 male mice were treated with 3 cycles of DSS alone or in combination with 8-strains probiotic or 9-strains probiotic (50*10^9 bacteria/kg/day). Pie charts showing the percentage distribution of bile acids in the fecal samples from mice in the different experimental groups. Graphs show mean ± SEM of 4 NT, 8 DSS, 5 DSS + 8-strains probiotic, and 5 DSS + 9-strains probiotic mice.

**Figure S3.** An example of an LCMS trace of the methanol extract of the bacterial mixture 9-strains probiotic supernatant, to which primary, secondary, and conjugated BAs have been added in culture broth. Along with the specifics of the multiple reaction monitoring (MRM) experiment and the associated peak area, each chromatographic trace displays the peaks of the HDCA, Δ5,6-LCA, 7-Keto-LCA, UDCA, tauro-alpha-Muricolic acid, alpha and beta Muricolic acids, tauro-HCA and HCA.
